# Recent advances in our understanding of the organization of dorsal horn neuron populations and their contribution to cutaneous mechanical allodynia

**DOI:** 10.1007/s00702-020-02159-1

**Published:** 2020-04-02

**Authors:** Cedric Peirs, Radhouane Dallel, Andrew J. Todd

**Affiliations:** 1 Université Clermont Auvergne, CHU Clermont-Ferrand, Inserm, Neuro-Dol, Clermont-Ferrand, F-63000 France; 2 Institute of Neuroscience and Psychology, College of Medical, Veterinary and Life Sciences, University of Glasgow, Glasgow, G12 8QQ UK

**Keywords:** Dorsal horn, Neurons, Cutaneous mechanical allodynia, Chronic pain

## Abstract

The dorsal horns of the spinal cord and the trigeminal nuclei in the brainstem contain neuron populations that are critical to process sensory information. Neurons in these areas are highly heterogeneous in their morphology, molecular phenotype and intrinsic properties, making it difficult to identify functionally distinct cell populations, and to determine how these are engaged in pathophysiological conditions. There is a growing consensus concerning the classification of neuron populations, based on transcriptomic and transductomic analyses of the dorsal horn. These approaches have led to the discovery of several molecularly defined cell types that have been implicated in cutaneous mechanical allodynia, a highly prevalent and difficult-to-treat symptom of chronic pain, in which touch becomes painful. The main objective of this review is to provide a contemporary view of dorsal horn neuronal populations, and describe recent advances in our understanding of on how they participate in cutaneous mechanical allodynia.

**Electronic supplementary material:**

The online version of this article (10.1007/s00702-020-02159-1) contains supplementary material, which is available to authorized users.

## Populations of dorsal horn neurons

### Neuronal composition of the dorsal horn

The spinal and medullary dorsal horns (DH) are the first central relay for somatosensory inputs innervating the extracephalic and trigeminal areas, respectively. Neuronal size and density vary along the dorso-ventral axis of the spinal DH (and the medio-lateral axis of the medullary DH), resulting in six parallel layers that are consistently found across mammalian species (Rexed 1952; Ribeiro-da-Silva and De Koninck 2008). Lamina I (a.k.a. the marginal layer) and lamina II (a.k.a. the substantia gelatinosa) form the superficial DH and appear translucent in living tissue, due to the low level of myelination in this area. The remaining DH laminae include the nucleus proprius (laminae III–IV), the neck (lamina V) and the base (lamina VI) of the DH. Nissl staining of the DH shows more numerous and smaller cells in laminae I–II compared to deeper laminae. Lamina I is a thin layer that includes cell bodies of both small and large neurons. It is distinct from lamina II, which contains densely packed neurons with small cell bodies. Lamina II is subdivided into two bands of approximatively equal size. In mice, the inner part of lamina II (II_i_) can be further subdivided into a dorsal (II_id_) and ventral (II_iv_) zone (Abraira et al. 2017). The border between lamina III and IV is usually set by the heterogeneity in neuronal size of lamina IV neurons, compared to the smaller cells of lamina III. Lamina V is marked by the presence of numerous myelinated afferents that form a reticulated area. Lamina VI, which only exists in the cervical and lumbosacral enlargements, is characterized by smaller and more regularly arranged cells than lamina V.

The great majority of spinal DH neurons have axonal arborizations that remain within the spinal cord and are thus considered interneurons (Bice and Beal 1997a). Many of them are intrasegmental, but some propriospinal interneurons can also send axons to other spinal segments (Bice and Beal 1997b; Gutierrez-Mecinas et al. 2018). DH interneurons make up to 95% of the total number of neurons in lamina I, virtually all neurons in lamina II (Bice and Beal 1997a; Spike et al. 2003) and around 98% of those in lamina III (Abraira et al. 2017). DH neurons display remarkable heterogeneity in morphology, electrophysiological properties and transcriptomic profiles (Gatto et al. 2019; Todd 2017), reflecting the complex role of the DH in integrating and modulating, rather than simply relaying, somatosensory inputs before they reach supraspinal regions. The different laminae of the DH are differentially enriched in neuropeptides and proteins (Abraira et al. 2017), suggesting the existence of populations of neurons that are organized in layers through the dorso-ventral axis to receive and process somatosensory information. Accordingly, the DH contains some neurons that are functionally highly specialized to process specific modalities or manifestations of pain (Koch et al. 2018). They also display complex cross-modality interactions (e.g., for inhibition or exacerbation of pain by touch) suggesting convergence rather than specificity in chronic pain circuits.

#### Excitatory versus inhibitory neurons

DH neurons can be classified into two major groups, based on their main neurotransmitter. In the DH, virtually all excitatory neurons are glutamatergic and express the vesicular glutamate transporter 2 (VGLUT2) (Oliveira et al. 2003; Todd et al. 2003). Inhibitory neurons release γ-aminobutyric acid (GABA) and/or glycine, although most glycinergic neurons in the superficial laminae are also thought to release GABA (Todd and Sullivan 1990). Interestingly, mice in which EGFP expression is driven by the glutamic acid decarboxylase 67 (GAD67) or the glycine transporter 2 (GLYT2) promoter revealed that GABAergic inhibitory neurons are preferentially expressed in laminae II–III, whereas those expressing glycine are located mostly in laminae III–V and lamina I (Zeilhofer et al. 2012b). Inhibitory neurons expressing GABA account for 30% of neurons in lamina I, 24% in lamina II and 38% in lamina III in the mouse DH, and similar proportions have been seen in the rat (Polgar et al. 2003, 2013a; Todd and Sullivan 1990). Importantly, analysis of the expression of transcription factors, mainly homeodomain (HD) and basic helix-loop-helix (bHLH), has revealed key elements that are involved in determining whether DH neuron display excitatory or inhibitory phenotypes (Lai et al. 2016). Some of these genes continue to be expressed in the adult, such as the transcription factor paired box gene 2 (PAX2), the gastrulation brain homeobox 1 (GBX1), the T-cell leukemia homeobox protein 3 (TLX3) and the LIM homeobox transcription factor 1 beta (LMX1B). This makes them suitable markers for the identification of inhibitory (PAX2, GBX1) and excitatory (TLX3, LMX1B) DH neurons (Del Barrio et al. 2013). Using such markers, it was recently shown in rats that 36–53% neurons in laminae I–III, and 54–58% of neurons in laminae IV–V are inhibitory neurons that coexpress PAX2 and γ-aminobutyric acid (GABA) (Larsson 2017).

#### Lamina I

**Fig. 1:**
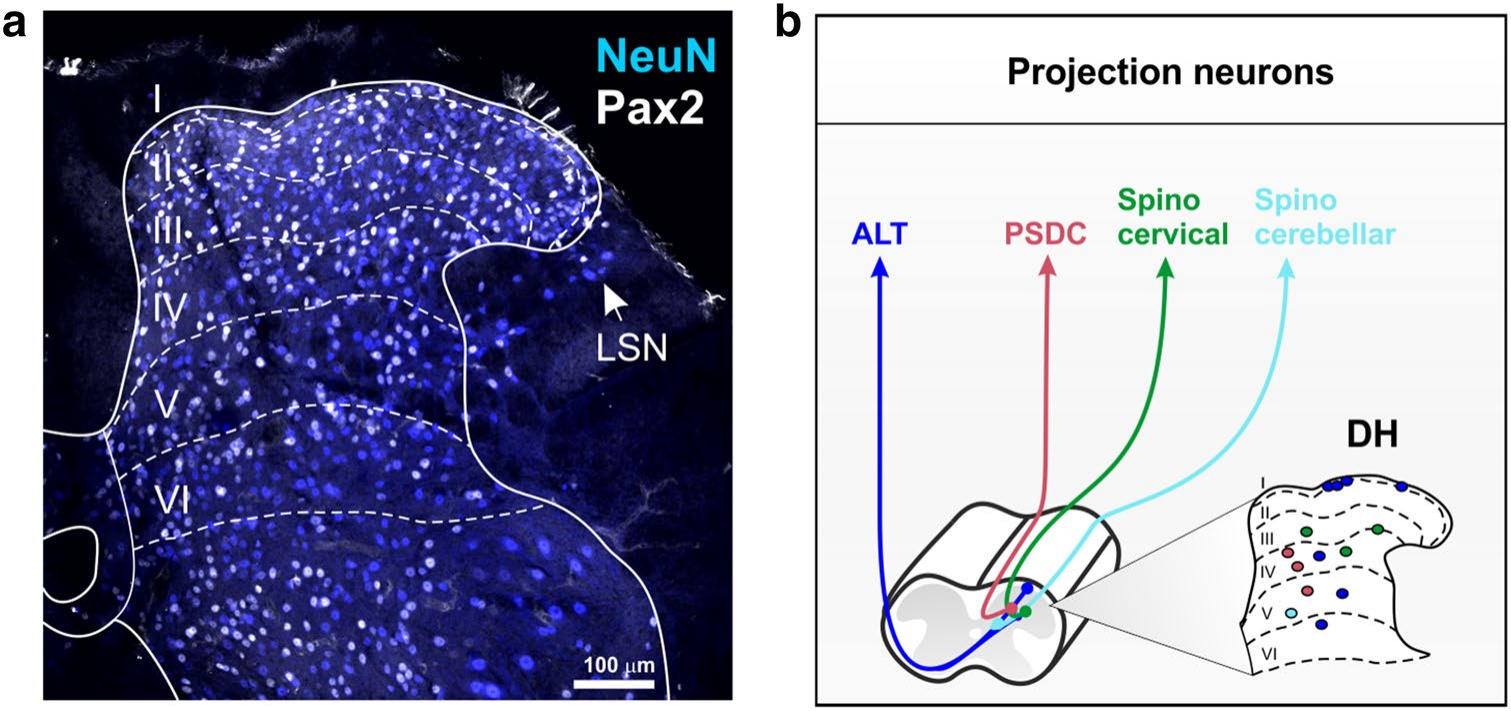
Spinal cord dorsal horn lamination. **a** Confocal image of a transverse section of mouse lumbar spinal cord immunostained with antibodies directed against NEUN to mark all neurons (blue), and against PAX2 to reveal only inhibitory neurons (white). Dotted lines represent boundaries of the six dorsal horn laminae. LSN, lateral spinal nucleus. Scale = 100 µm. **b** Schematic of the location and trajectories of projection neurons within the dorsal horn. *ALT* antero-lateral tracts, *PSDC* post-synaptic dorsal columns.

For over a century since the work of Ramón y Cajal (1909), several attempts have been made to classify neuronal populations using cell morphology, with the hope that cellular shape would relate to specific functions in sensory processing. Early Golgi staining of the DH revealed neurons in lamina I with pyramidal, multipolar and fusiform morphologies (Lima et al. 1993; Lima and Coimbra 1986; Zhang et al. 1996) (Fig. 1, Online Resource 1 and 4). Pyramidal cells have triangular perikarya in any viewing plane, with dendrites that typically remain in lamina I in the medio-lateral axis. Multipolar cells have round perikarya with dendrites emerging in various directions, whereas fusiform cells have typical elongated perikarya in the rostro-caudal axis with elongated longitudinal bipolar dendrites, but round cell bodies in transverse sections. These studies, however, did not distinguish interneurons from projection neurons, meaning that most of these morphological classes are likely to include both types, and are therefore distributed among distinct functional populations.

Interestingly, there is a correlation between lamina I cell morphology and their respective pattern of action potential discharge. Upon current injection during patch-clamp recording, lamina I neurons can be identified as tonic—which fire slowly but continuously, phasic (a.k.a. adapting or initial bursting)—which fire with a high frequency burst of variable duration, delayed—which fire with a marked delay to the first spike, and single spike—which fire a single action potential even upon strong depolarization (Prescott and De Koninck 2002). Fusiform neurons are typically tonic, pyramidal ones are phasic, and multipolar cells are either delayed or single spiking. Importantly, these firing patterns seem to be related to specific functions within the DH, such as tonic and delayed cells that predominantly act as integrators, whereas phasic and single spike cells serve as coincidence detectors (Prescott and De Koninck 2002).

##### Projection neurons

Among lamina I neurons, a subset comprises projection cells that send axons through the anterolateral tracts (ALT) in the contralateral spinal cord, to the lateral parabrachial nucleus (LPb), the caudal ventrolateral medullary reticular formation (CVLM), the periaqueductal gray (PAG) and the thalamus (Al-Khater and Todd 2009; Spike et al. 2003). Very little is known about the molecular phenotype of ALT lamina I neurons, as most if not all molecular markers identified in lamina I are also found in deeper laminae (Koch et al. 2018). Early work identified several neuropeptides in lamina I neurons following colchicine treatment to increase the concentration of peptides in the cell bodies (Willis and Coggeshall 1978). It is however unclear whether these are expressed in physiological conditions, as colchicine can alter mRNA expression, with potential de novo expression of peptides (Cortes et al. 1990). It has been estimated that 80% and 90% of ALT lamina I neurons express the neurokinin 1 receptor (NK1R) in rats and mice, respectively (Al Khater et al. 2008; Cameron et al. 2015; Spike et al. 2003; Todd et al. 2000). As suggested above, these include pyramidal, multipolar and fusiform types (Almarestani et al. 2007; Brown et al. 1995; Polgar et al. 2008; Spike et al. 2003; Zhang et al. 1996). NK1R + ALT lamina I neurons are generally larger than surrounding interneurons (Al Ghamdi et al. 2009), making the large size of NK1R + neurons a relatively good indicator for identifying projection neurons in lamina I. Around 20% of NK1R + lamina I neurons express the gamma isoform of protein kinase C (PKCγ) (Polgar et al. 1999a), and these typically have a fusiform morphology (Polgar et al. 1999a). However, the NK1R is not restricted to projection neurons, but is also expressed at a lower level in some interneurons throughout the DH (Polgar et al. 2013a; Todd et al. 1998). PKCγ is expressed by the majority of trigeminothalamic neurons in lamina I of the medullary DH (Li et al. 2001). There is a small population of giant multipolar neurons that are densely coated with inhibitory and excitatory synapses. These cells, which generally lack the NK1R, account for about 3% of ALT lamina I neurons in the rat (Polgar et al. 2008; Puskar et al. 2001). A small fraction of trigeminothalamic neurons in lamina I express dynorphin (DYN) (Li et al. 1999). Interestingly, ALT lamina I projection neurons seem to display different firing patterns compared to other neurons in this area (Ruscheweyh and Sandkuhler 2002), with sustained rhythmic discharge with either constant interspike intervals, or bursts of high-frequency action potentials (Grudt and Perl 2002; Luz et al. 2014, 2019; Ruscheweyh et al. 2004).

##### Interneurons

As with projection neurons, knowledge about the phenotype of lamina I interneurons is limited (Koch et al. 2018). Lamina I contains scattered neurons belonging to larger populations that extend into deeper laminae. These include neurons expressing calretinin (CR) (Gutierrez-Mecinas et al. 2019c), substance P (SP) (Gutierrez-Mecinas et al. 2018), cholecystokinin (CCK) (Gutierrez-Mecinas et al. 2019b) or parvalbumin (PV) (Laing et al. 1994), which are often not quantified, or make up a very small fraction of neurons in this area. Excitatory neurons that express preprotachykinin (PPTB), the precursor for neurokinin B (NKB), make up ~ 11% of all lamina I neurons in the rat (Polgar et al. 2006), although these cells are not seen in the mouse (Gutierrez-Mecinas et al. 2016). Additionally, PKCγ + neurons are exclusively excitatory in lamina I and account for 8% of all neurons in this area, including the small fraction of ALT neurons mentioned above (Peirs et al. 2014; Polgar et al. 1999a). Lamina I DYN + cells do not express PKCγ and are presumably mostly interneurons (Marvizon et al. 2009), aside from the few trigeminothalamic neurons mentioned above. They make up about 17% of lamina I neurons in the rat (Sardella et al. 2011a). The DYN + neuron population, however, is heterogeneous, as about 50% of lamina I DYN + neurons express GABA and account for about 32% of lamina I inhibitory neurons in this species (Sardella et al. 2011a). The remaining lamina I DYN + neurons are presumably excitatory neurons that do not express NK1R (Marvizon et al. 2009). Interestingly, in the mouse (but not in the rat) the excitatory DYN + population is located almost exclusively in regions of the DH that receive innervation from glabrous skin (Boyle et al. 2017). Entirely included within the lamina I DYN + inhibitory population are neurons expressing galanin (GAL) (Sardella et al. 2011a) which are virtually all GABAergic (Simmons et al. 1995). A distinct population of inhibitory neurons in lamina I express neuropeptide Y (NPY) and these make up around a quarter of the inhibitory neurons in this lamina (Polgar et al. 2011). These are largely distinct from neurons that belong to the DYN + population (Boyle et al. 2017). Additionally, neurons expressing the neuronal nitric oxide synthase (NNOS) are virtually all GABAergic in lamina I. In the rat, these are distinct from DYN + and NPY + neurons, and account for 17% of inhibitory neurons in this area (Laing et al. 1994; Sardella et al. 2011b). A subset of small DH lamina I cells express the gastrin-releasing peptide receptor (GRPR), but not NK1R (Sun et al. 2009), and these seem to mediate itch but not pain (Sun and Chen 2007). Because of their small cell bodies, these are likely interneurons, and different from lamina I spinothalamic neurons identified in cats that preferentially mediate itch (Andrew and Craig 2001).

#### Lamina II interneurons

**Fig. 2:**
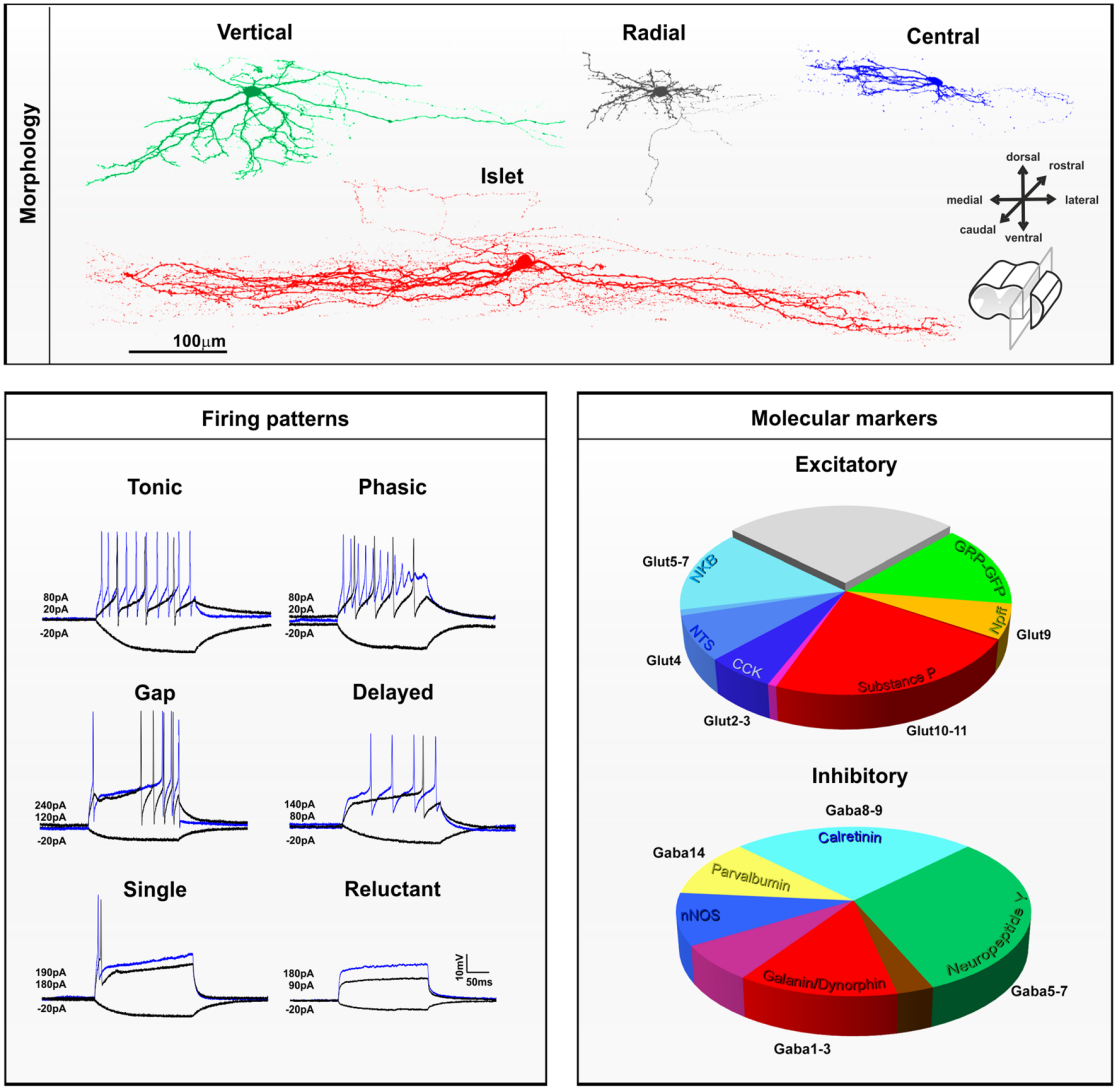
Morphological, electrophysiological and neurochemical features of interneurons in laminae I–II of the mouse dorsal horn. *Top panel* Morphological features of lamina II dorsal horn neurons observed in sagittal slices. Confocal images of neurons filled with neurobiotin showing vertical (green), radial (gray), central (blue) or islet (red) morphologies. Scale = 100 µm. *Lower left panel* Electrophysiological whole-cell patch-clamp recording of dorsal horn neurons. Firing pattern of dorsal horn neurons can be tonic, phasic, with a gap between spikes, or with one or no action potential upon depolarizing current injection. Traces in black display membrane potential at -20pA or at rehobase respectively. Superimposed blue traces are representative firing patterns observed at suprathreshold current injection. Value for hyperpolarizing and depolarizing currents are indicated. *Lower right panel* The proportions of excitatory and inhibitory interneurons in this region that belong to different neurochemical populations. The relationship to the different transcriptomic populations identified by Haring et al. (2018) is also shown. Note that the *NKB* neurokinin B, *NTS* neurotensin, *CCK* cholecystokinin, *SP* substance P, *NPFF* neuropeptide FF and GRP–GFP cells form largely non-overlapping populations of excitatory interneurons, although there is some overlap between NKB/NTS and CCK/SP populations [reproduced from Gutierrez-Mecinas et al. (2019a)]. The GRP–GFP cells are defined as those that express GFP in the BAC transgenic GRP::eGFP line. For inhibitory interneurons, there is overlap between the galanin/dynorphin (GAL/DYN) population and the neuronal nitric oxide synthase (NNOS) population, and this is shown in purple. Similarly, the GAL/DYN population overlaps with the NPY population, and this is shown in brown. There is limited overlap between NPY cells and both NNOS and parvalbumin (PV) cells, although this is not shown on the pie chart. Reproduced from Boyle et al. (2017)

Most investigations of DH neurons have been performed in lamina II (Merighi 2018), the core of the gate control theory, which postulated an interaction between nociceptive and non-nociceptive inputs at the spinal level (Melzack and Wall 1965). In this proposal, inhibitory interneurons in lamina II are activated by non-nociceptive sensory neurons to reduce pain. However, when DH inhibition is diminished after injury, innocuous mechanical stimulation of the skin no longer reduces DH nociceptive activity, but rather engages nociceptive circuits through a dorsally directed polysynaptic pathway, leading to cutaneous mechanical allodynia (CMA) (Braz et al. 2014). In lamina II, the broadly accepted morphological classification of Grudt and Perl (2002) identified vertical, radial, central and islet cells (Fig. 2, Online Resource 2 and 4). Importantly, the original description of these morphologies was specific to neurons in this area, and obtained in sagittal slices, which include most of their dendritic arbors (Lu and Perl 2005; Punnakkal et al. 2014; Todd and Lewis 1986). Lamina II vertical, radial, central and islet cells, which will be briefly described below, are thus quite distinct from other DH neurons described across the dorso-ventral axis, and yet these terms are often assigned to neurons outside lamina II, or to neurons observed in transverse slices (Koch et al. 2018). Nevertheless, the following section will classify lamina II neurons based on the Grudt and Perl scheme, including current knowledge of their molecular and electrophysiological profiles. Indeed, as in lamina I, DH lamina II neurons can be distinguished depending on their pattern of action potential discharge. However, in addition to be tonic, phasic, delayed or single spike, lamina II neurons also include gap (a.k.a. irregular)—which have a delay between two spikes that is greater than 1.5 times the delay between two previous or two following spikes, and reluctant (which do not spike upon depolarization current) neurons (Balachandar and Prescott 2018).

##### Vertical cells

Lamina II vertical cells, previously described as “stalked neurons”, have extensive dendrites running ventrally in a cone shape through the dorso-ventral axis, when viewed in sagittal slices (Gobel 1978; Maxwell et al. 2007). Vertical cells are thought to represent a population of excitatory DH neurons with cell bodies located mostly in the outer part of lamina II, and include cells that send axons directly to lamina I projection neurons (Boyle et al. 2019; Cordero-Erausquin et al. 2009; Lu et al. 2013; Lu and Perl 2005). It has been recently suggested that lamina II interneurons expressing DYN include vertical cells (Duan et al. 2014) and belong to the excitatory component of the otherwise mixed lamina II DYN + population (Huang et al. 2018). A subset of excitatory neurons expressing CR may also include vertical cells (Smith et al. 2015, 2016). Although it was reported that cells expressing green fluorescent protein under the control of the promotor for gastrin-releasing peptide (GRP), included vertical cells (Sun et al. 2017), these had dorsally directed dendrites and therefore do not fit the Grudt and Perl criteria of vertical cells. Vertical cells have tonic or delayed firing patterns and invariably show sustained action potentials during a depolarizing step, with regular or irregular interspike intervals (Grudt and Perl 2002). These particular firing properties have been reported in the putative vertical cells described above, including delayed firing lamina II excitatory DYN + (Huang et al. 2018) and CR + neurons (Smith et al. 2015), but are rarely seen in GRP + neurons (Dickie et al. 2018).

##### Radial cells

Lamina II radial cells have relatively short dendrites radiating in all directions, as seen in both transverse and sagittal sections. However, the term has also been applied to “stellate cells” described in humans, which are multipolar neurons with straight dendrites that cover a very large elliptic area, extending up to 500 µm into laminae I and III (Schoenen 1982). Radial cells described by Grudt and Perl are, however, quite distinct, with small and highly branched dendrites that do not extend more than few tens of micrometers in the dorso-ventral or medio-lateral axis, and less than 200 µm in the rostro-caudal plane. Axons of radial cells are generally located in lamina II, with some running in the dorsolateral fasciculus to target the lateral spinal nucleus (LSN) (Dickie et al. 2018). It has recently been reported that excitatory neurons in lamina II expressing substance P (SP) (Dickie et al. 2018), and some of those expressing PKCγ (Abraira et al. 2017; Alba-Delgado et al. 2015) or CR (Smith et al. 2015, 2016), correspond to the radial cells defined by Grudt and Perl. Radial cells all have delayed firing patterns, with irregular interspike intervals or high-frequency bursts (Grudt and Perl 2002). Accordingly, the large majority of CR + (Smith et al. 2015) and SP + (Dickie et al. 2018) neurons, and most PKCγ + (Abraira et al. 2017) neurons, have delayed firing, except in the medullary DH where radial PKCγ + neurons are never delayed (Alba-Delgado et al. 2015).

##### Central and islet cells

Lamina II central and islet cells have characteristic dendrites that are elongated in the sagittal plane and can extend, for islet cells, to considerable distances (> 400 µm in the rat) in the rostro-caudal axis. Because of this particular spatial orientation, identification of these cells is difficult or impossible in transverse sections, particularly for islet cells (Gobel 1975; Maxwell et al. 2007; Smith et al. 2015). Central cells can have either tonic or phasic firing patterns (Grudt and Perl 2002). A specific population of excitatory interneurons, originally described as “transient central cells” (Lu and Perl 2005), are thought to receive input from PKCγ + neurons and to project directly to vertical cells (Lu et al. 2013). It has been reported that lamina II excitatory GRP + neurons (Albisetti et al. 2019; Dickie et al. 2018) and subpopulations of excitatory neurons in inner lamina II that express PKCγ (Alba-Delgado et al. 2015) or CR (Smith et al. 2015, 2016) show central morphology. Accordingly, the majority of GRP + (Dickie et al. 2018; Pagani et al. 2019) and a third of PKCγ + (Abraira et al. 2017) neurons are phasic, with an initial burst of one or more action potentials, except for trigeminal central PKCγ + neurons, which are mostly tonic (Alba-Delgado et al. 2015). Some excitatory CR + neurons, which include cells with central morphology, also show phasic firing properties in addition to their characteristic delayed first action potential (Smith et al. 2015). Interestingly, the majority of lamina II excitatory GRP + , CR + or PKCγ + neurons that are not delayed and/or phasic fall into the class of single spiking or reluctant spiking neurons (Abraira et al. 2017; Dickie et al. 2018; Smith et al. 2015), and this may reflect an extreme form of adaptation that characterizes the phasic firing pattern. Surprisingly, in the spinal DH, excitatory lamina II GRP + , CR + or PKCγ + neurons are rarely or never tonic, indicating that tonic central cells, which account for about a quarter of recorded central cells (Grudt and Perl 2002), might represent a distinct functional class of inhibitory cells. Lamina II inhibitory neurons expressing neuropeptide Y (NPY) (Iwagaki et al. 2016), parvalbumin (PV) (Abraira et al. 2017), or CR (Smith et al. 2015) are nearly all tonic, but only the PV + population includes cells with central morphology.

Islet cells are invariably inhibitory interneurons and use either GABA, glycine or both neurotransmitters (Heinke et al. 2004; Maxwell et al. 2007; Todd and McKenzie 1989; Todd and Sullivan 1990; Yasaka et al. 2010). Interestingly, the islet cell population does not include NPY + inhibitory neurons (Iwagaki et al. 2016). Islet cells include inhibitory neurons that express PV (Abraira et al. 2017; Boyle et al. 2019) or CR (Smith et al. 2015, 2016), the latter population accounting for up to 15% of the entire CR + population, and including a group of inhibitory neurons that express preprotachykinin A (PPTA), the precursor for SP (Gutierrez-Mecinas et al. 2019c; Smith et al. 2015, 2016). PV + inhibitory neurons innervate PKCγ + neurons (Petitjean et al. 2015) and also provide axo-axonic synapses onto myelinated LTMR afferent terminals (Boyle et al. 2019). The target of the axons of CR + inhibitory neurons is unknown, but as islet cells they most likely reside in the same layer as their cell bodies (Abraira et al. 2017; Smith et al. 2016). Notably, all islet cells have tonic firing properties (Grudt and Perl 2002). However, it is important to note that inhibitory neurons are not always islet cells and do not always show tonic firing. For example, inhibitory cells that express galanin and dynorphin (GAL/DYN), NNOS or NPY, are morphologically and electrophysiologically heterogeneous (Ganley et al. 2015; Iwagaki et al. 2016, 2013).

##### Non-overlapping populations of lamina II neurons

Interestingly, lamina II neurons can be classified based on non-overlapping expression of different neurochemical markers, regardless of their morphology or intrinsic firing properties (Fig. 2). Among the excitatory neurons in laminae I–II, six largely non-overlapping populations can be defined based on expression of neurotensin (NTS), SP (also referred to as Tachykinin 1 (TAC1) or PPTA), NKB (also referred to as TAC2 or PPTB), GRP, cholecystokinin (CCK) or neuropeptide FF (NPFF). In this scheme, the GRP cells are defined by the expression of enhanced green fluorescent protein (EGFP) in a bacterial artificial chromosome (BAC) transgenic mouse line (GRP::EGFP). Although all of the EGFP cells in this mouse line possess the mRNA for GRP, they seem to represent a distinct subset among the GRP + neurons (Gutierrez-Mecinas et al. 2019a). These neurochemically defined populations account for 9% (NTS), 24% (SP), 14% (NKB), 15% (GRP), 7% (CCK) and 6% (NPFF) of all excitatory laminae I–II neurons in the mouse (Gutierrez-Mecinas et al. 2016, 2017, 2019a, b; Mar et al. 2012), accounting for about 75% of all excitatory neurons in this area. The remaining 25% of lamina II excitatory neurons are likely to include vertical cells, which are rarely found within these six populations of cells. Although vertical cells have been described among certain neurochemical classes, such as those expressing somatostatin (SOM) (Duan et al. 2014), enkephalin (ENK) (Francois et al. 2017) or CR (Gutierrez-Mecinas et al. 2019c), these classes are relatively broad and overlap extensively with each other, as well as with the six classes defined above. For example, many excitatory neurons in laminae I–II contain CR, and these seem to correspond to those that express SP, NKB, as well as many of the GRP-EGFP cells, but not those that express NPFF, CCK or NTS (Gutierrez-Mecinas et al. 2019c; Haring et al. 2018).

For inhibitory interneurons in this region, a similar approach has revealed five largely non-overlapping populations that express GAL/DYN, NPY, NNOS, PV or CR. These populations account for nearly all laminae I–II inhibitory neurons, making up to 24% (GAL/DYN), 33% (NPY), 17% (NNOS), 11% (PV) and 27% (CR) of the inhibitory cells in this area (Boyle et al. 2017; Gutierrez-Mecinas et al. 2016, 2017, 2019c). Importantly however, in contrast to NPY + lamina II neurons, which are nearly all inhibitory (Rowan et al. 1993), neurons that express DYN (Sardella et al. 2011a), NNOS (Sardella et al. 2011b), PV (Abraira et al. 2017; Laing et al. 1994) or CR (Gutierrez-Mecinas et al. 2019c; Smith et al. 2015) include a relatively large proportion of excitatory cells. Neurons that express the basic helix-loop-helix domain containing, class B, 5 (BHLHB5) during development have been implicated in the inhibition of itch, and these include the vast majority of the GAL/DYN + and NNOS + populations (Kardon et al. 2014). However, it was recently shown that in contrast to the GAL/DYN + population which suppresses pruritogen-evoked itch, NNOS + neurons are likely to have an anti-nociceptive role (Huang et al. 2018).

How this classification of excitatory and inhibitory interneurons fits with specific functions in chronic pain processing is under investigation (Todd 2017).

#### Laminae III–VI

Laminae III–IV are usually considered together, not from a functional perspective, but because neurons in this area have dendritic trees that generally extend within these laminae, and because cells within them give rise to several ascending tracts (Brown 1982). Deep DH neurons include both projection neurons and interneurons with several morphological patterns and axons that typically run in the ventral direction (Willis and Coggeshall 1978) (Fig. 1, Online Resource 3 and 4). Dendritic trees usually form conical arborizations in laminae III–IV, but are better described as flattened disks in laminae V–VI. They extend in both rostro-caudal and dorso-ventral directions in lamina III, but are mostly restricted to the transverse plane in deeper laminae (Scheibel and Scheibel 1968). A subset of lamina III neurons are antenna-type cells with long dorsally directed dendrites, some of which express NK1R (Fernandes et al. 2018; Naim et al. 1997). In the rat, these NK1R + cells can be identified as projection cells belonging to the ALT (Marshall et al. 1996; Naim et al. 1997; Todd et al. 2000). However, other cells with similar morphology that lack the NK1R appear to be interneurons (Polgar et al. 2007). Within the deep DH, there are several neurons that synapse directly onto ventral horn motor neurons. It is important to note that these pre-motor neurons are located through the whole DH, but are highly concentrated in the lumbar laminae IV–VI. These neurons were recently identified as excitatory and inhibitory interneurons that express the transcription factor TCFAP2β and the nuclear and chromatin organization factors SATB1/2 during development, and are located predominantly in medial lamina V (Levine et al. 2014). The firing patterns of lamina III–IV neurons are similar to those in lamina II, and include tonic, phasic, delayed, single, gap and reluctant spiking (Abraira et al. 2017). In contrast, cells in the deepest part of the DH have mostly tonic or phasic firing patterns, with higher firing frequency than in superficial laminae (Ruscheweyh and Sandkuhler 2002). Of note, medullary DH neurons located in lamina V also include neurons with delayed firing (Morisset and Nagy 1998).

##### Projection neurons

The deep DH contains several classes of projection neurons. These give rise to various tracts, but most of them send projections through the spinocervical tract (SCT), the ALT, or the dorsal columns (Brown 1982). Laminae III–IV SCT neurons send axons through the most medial and superficial parts of the ipsilateral dorsal funiculus (Brown 1982). These cells usually have elongated dendrites that form a cylinder in the dorso-ventral axis. However, it is unclear whether the SCT exists in humans, as this tract might be rudimentary in primates (Truex et al. 1970).

Lamina III–IV cells include ALT projection neurons that send axons to the thalamus, CVLM and LPb area. The proportion of spinothalamic DH neurons in these laminae is highly dependent on the spinal segment, and these are numerous in the cervical and thoracic spinal cord, but much less frequent in the lumbosacral region (Al Khater et al. 2008; Burstein et al. 1990; Davidson et al. 2010). In contrast to lamina I spinothalamic cells described above, most of those in the deep DH are located in laminae V–VI and have very large cell bodies and dendritic trees spreading across multiple laminae (Willis et al. 1979). They display several morphologies with dendrites oriented dorsally up to lamina I, or radiating as far as the lateral funiculus or the border of laminae VII and X. There are also a few neurons of the spinoreticular (SRT) and spinomesencephalic (SMT) tracts in the lateral part of laminae V–VI with multipolar morphologies and long straight dendrites (Kevetter et al. 1982; Menetrey et al. 1982). Axons of lamina III ALT neurons typically cross the ventral commissure near their cell body and run mostly through the ventrolateral quadrant, and also through the dorsolateral funiculus in cats, rats and monkeys (Sengul and Watson 2015). Two major sources of local synaptic input to lamina III ALT cells have been identified, originating from DYN + excitatory and NPY + inhibitory neurons (Baseer et al. 2012; Naim et al. 1997; Polgar et al. 1999b). In the rat, the great majority of these lamina III ALT projection neurons show strong NK1R expression (Ding et al. 1995; Marshall et al. 1996; Todd et al. 2000). A similar population of ALT neurons is also present in the mouse, but in this species, few of them express NK1R (Cameron et al. 2015).

Laminae III–V include neurons that belong to the postsynaptic dorsal column (PSDC) system, which send axons to the ipsilateral gracile or cuneate nucleus through the dorsal columns (Brown 1982). They are located in the medial part of the DH with extensive dendritic trees that radiate in various axes, but that are mainly restricted to the transverse plane (Brown and Fyffe 1981). The molecular identity of PSDC cells is unclear, but some of these cells transiently express the transcription factor ZIC2 (Paixão et al. 2019). Interestingly, these cells do not appear to express the NK1R (Polgar et al. 1999b, 2007).

Additionally, most neurons of the spinocerebellar tract are located in the ventral horn, mainly in the Clarke’s columns, but a few of them have also been identified in the DH lamina V, particularly in the thoracic and the rostral half of the lumbar spinal cord (Matsushita and Hosoya 1979). Interestingly, in contrast to spinocerebellar neurons from Clarke’s column, which selectively express lial derived neurotrophic factor (GDNF) and send axons through the contralateral spinal cord (Hantman and Jessell 2010), DH spinocerebellar neurons run through the ipsilateral lateral funiculus (Edgley and Gallimore 1988). These cells have recently been identified as deep DH excitatory neurons that express the basic helix-loop-helix (bHLH) transcription factor, ATOH1 (Yuengert et al. 2015). These cells are critical to perform motor tasks, but are dispensable for nocifensive pain behavior.

Several of these projection neurons have been quantified in the deep DH, but the relative proportion of each of these cell classes within this region is not available. The molecular phenotype and firing patterns of these projection neurons are also unclear, but they may belong to excitatory populations of the deep DH including those expressing LMX1B, the ladybird homeobox I (LBX1), the ROR alpha nuclear orphan receptor (RORα), RORβ, the V-maf musculoaponeurotic fibrosarcoma oncogene homolog A (MAFA), MAFB or CMAF (Del Barrio et al. 2013), and may feature in recent DH single-cell transcriptomic studies (Haring et al. 2018; Sathyamurthy et al. 2018; Zeisel et al. 2018).

##### Excitatory interneurons

As mentioned above, the deep DH contains both projection neurons and interneurons. The morphology of deep DH interneurons is highly heterogeneous and has been described for several populations of neurochemically defined cells. Some deep DH neurons were suggested to have morphologies similar to vertical, radial or central cells described by Grudt and Perl in lamina II, such as excitatory neurons in lamina III/IV expressing RORα (Bourane et al. 2015b) or those transiently expressing VGLUT3 (Peirs et al. 2015). Once again, while it is likely that those cells do belong to real morphological classes, they seem to be distinct from lamina II neurons, with different morphology and neurochemistry. The neurochemical populations identified in this region are also not exclusive to the deep DH. About 60% of RORα + neurons also express CCK (Bourane et al. 2015b), and RORα + neurons also partially overlap with the excitatory subset of neurons that express RORβ (Del Barrio et al. 2013). As indicated earlier, a few scattered excitatory neurons in lamina III express NK1R or PKCγ and are included in the major cell populations of the deep DH that express SOM (Duan et al. 2014), CCK (Gutierrez-Mecinas et al. 2019b), RORα (Bourane et al. 2015b) or VGLUT3 during development (Peirs et al. 2015). SOM + neurons are predominantly excitatory neurons with phasic, delayed and single spiking patterns, and account for 16–17% of lamina III neurons (Duan et al. 2014; Gutierrez-Mecinas et al. 2016). CCK + excitatory neurons, which again partially overlap with the excitatory RORα + and RORβ + cells, account for a third to a quarter of excitatory neurons in lamina III (Gutierrez-Mecinas et al. 2019b) and have phasic or tonic firing patterns (Abraira et al. 2017). VGLUT3 + neurons are subdivided into at least two populations, one with tonic or phasic firing patterns (Peirs et al. 2015), and a second located more dorsally that predominantly displays delayed firing (Cheng et al. 2017).

##### Inhibitory interneurons

The deep DH contains large populations of inhibitory neurons such as those expressing the receptor tyrosine kinase Ret (RET) (Cui et al. 2016) and some that express Rorβ (Koch et al. 2017). Based on the morphological classification described in lamina II by Grudt and Perl, it has been suggested that these neurons have morphologies similar to vertical, radial and islet cells for the RET + population, and vertical and central cells for the RORβ + population, with the exception of RORβ + neurons of laminae V–VI that resemble islet cells. Similar to the excitatory neurons described above, the morphologies of these cells are, however, most likely unrelated to those assigned to lamina II neurons, although they may reflect specific functions of neurons of the deep DH. In contrast to inhibitory neurons in lamina II described above which mostly have tonic firing patterns, those in lamina III include tonic, phasic, delayed, gap and reluctant firing (Abraira et al. 2017). Tonic firing is, however, still predominant in the deep DH, in both GABAergic and glycinergic neurons (Punnakkal et al. 2014). Similar to the excitatory neuron populations of the deep DH described above, inhibitory neurons in this area are highly heterogeneous. RET + inhibitory neurons make up about one-third of all inhibitory neurons of the deep DH and these cells express other inhibitory markers such as GLYT2, GAD1/2 and PV, but not NNOS or DYN (Cui et al. 2016). As for excitatory neurons of the deep DH, scattered inhibitory neurons in this area express markers described in superficial laminae, such as NPY, DYN/GAL, NNOS, PV or CR (Gutierrez-Mecinas et al. 2019c; Polgar et al. 2013a, b; Sardella et al. 2011a). However, in contrast to the superficial laminae, inhibitory neurons in lamina III do not apparently coexpress GAL and DYN (Sardella et al. 2011a). In fact, DYN + neurons make up less than 1% of lamina III neurons and include excitatory neurons in the medial part of the DH in this area (Boyle et al. 2017). GAL + neurons in lamina III coexpress NNOS and account for 5% of inhibitory neurons in this area (Tiong et al. 2011). The firing pattern of these cells is unknown. PV + inhibitory neurons make up about 8% of all lamina III neurons (Abraira et al. 2017). Because about 40% of lamina III neurons express GABA, we can estimate that the PV + inhibitory population represents 20% of inhibitory neurons in this area (Polgar et al. 2013a). PV + inhibitory neurons have rostro-caudally elongated dendrites, resembling lamina II islet cells (Abraira et al. 2017; Boyle et al. 2019; Hughes et al. 2012) and generally show tonic firing (Abraira et al. 2017; Boyle et al. 2019; Hughes et al. 2012). CR + inhibitory neurons are very sparse in lamina III, but more numerous in laminae IV–VI. Most of them express PAX2 (Peirs, unpublished observation) but not much is known about their relative number or firing patterns. Similar to lamina II, NPY + inhibitory neurons in lamina III do not express DYN/GAL, PV or NNOS (Iwagaki et al. 2016). NPY + inhibitory neurons account for up to 25% of inhibitory neurons in lamina III (Boyle et al. 2017) and are morphologically heterogeneous, but never display elongated dendrites like islet cells (Iwagaki et al. 2016). Lamina III NPY + inhibitory neurons are mostly tonic firing neurons, but also include phasic and single spiking cells (Iwagaki et al. 2016).

##### Non-overlapping populations of deep dorsal horn neurons

Interestingly, a recent study used the Allen Brain Institute Spinal Cord Atlas to identify seven classes of excitatory and four classes of inhibitory DH neurons in lamina II_i_–III that largely do not overlap, and that account for 70–82% of all neurons in this area (Abraira et al. 2017). This classification includes excitatory neurons expressing cerebellin-2 (CBLN2), CCK, the serotonin receptor 6 (5HTR6), the insulin-like growth factor binding protein 5 (IGFBP5), the neurogenic differentiation factor-4 (NEUROD4), PV or PKCγ, and inhibitory neurons expressing cadherin-3 (CDH3), the Kv channel interacting protein-2 (KCNIP2), RORβ or PV. Of note, this classification of DH neurons partially matches previously described cell populations, such as deep excitatory neurons expressing CCK that have recently been implicated in touch processing (Abraira et al. 2017; Liu et al. 2018), or deep inhibitory neurons expressing PV that contribute to feedforward inhibition of peripheral sensory neurons and DH interneurons (Boyle et al. 2019; Petitjean et al. 2015).

### Classification of dorsal horn neuron populations: pitfalls and future directions

The DH clearly displays a high degree of heterogeneity in neuronal morphologies, neurochemistry and intrinsic properties. Studies described above that performed morphometric analysis of neurochemicaly identified DH neurons have shown that a morphological class is often shared by several cell subpopulations, including by projection neurons and interneurons, or excitatory/inhibitory interneurons classes, thus making it difficult to determine how morphology or neurochemistry relates to function, and what criteria define a functionally distinct population of cells. A good example of such complexity can be seen for one of the DH populations first implicated in CMA: PKCγ-expressing cells. These are nearly exclusively excitatory interneurons (Polgar et al. 1999a) and make up 30% of neurons in the inner part of lamina II, with very scattered cells in laminae III and I (Mermet-Joret et al. 2017; Peirs et al. 2014). Such a restricted cellular phenotype and location within the DH make these cells good candidates to define a functionally distinct population of DH neurons, and these neurons have indeed been the subject of numerous investigations (Artola et al. 2020). However, morphological (Peirs et al. 2014), electrophysiological (Alba-Delgado et al. 2015) and transcriptomic (Haring et al. 2018) analysis of PKCγ + neurons revealed significant heterogeneity in these cells, exposing at least two subgroups of PKCγ + neurons, with potentially distinct functions. In fact, PKCγ + neurons can be distributed among the non-overlapping populations of DH neurons described above. Cells in laminae II–III that possess PKCγ frequently coexpress NTS (Polgar et al. 1999a), CCK (Gutierrez-Mecinas et al. 2019b) or NKB (Gutierrez-Mecinas et al. 2016), although the NKB + cells generally show very low levels of the kinase. It has recently been reported that most (~ 85%) of the cells with strong PKCγ immunoreactivity express either NTS or CCK (Gutierrez-Mecinas et al. 2019b; Polgar et al. 1999a), and it will be of interest to determine whether these groups correspond to the subclasses of PKCγ neurons that were previously identified. Furthermore, as mentioned above, a significant proportion of ALT lamina I neurons express PKCγ, suggesting again that expression of the kinase alone is not sufficient to define a unique neuronal population.

It is also important to note that regardless of the location within the DH, there are several neurons described in the above studies that could not be assigned to any of the morphological classes that have been proposed, once again making it difficult to build a bridge between DH cell morphology, molecular phenotype and function. Remarkably, however, analysis of laminae II_i_–IV DH neurons has recently demonstrated that linear discriminant morphometric analysis of DH neurons, including cell body size, neurite length, spine density and neurite complexity using Sholl-based metrics and branching index measurements, creates linear classifiers that recognize interneuron subtypes with up to 88% accuracy (Abraira et al. 2017). Of note, however, it is important to emphasize that morphological features identified in healthy DH neurons may no longer apply after injury. For example, the population of PKCγ + neurons described in Abraira et al. (2017) have a dendritic tree that severely shrinks after peripheral inflammation (Alba-Delgado et al. 2018).

Importantly, recent clustering from single cell transcriptomic analysis of the spinal cord has revealed about 15 populations of DH excitatory and 15 populations of DH inhibitory neurons (Haring et al. 2018; Sathyamurthy et al. 2018; Zeisel et al. 2018). Notably, most clusters are defined by coexpression of more than one marker, which most likely better reflects the DH complexity compared to previous DH neuronal classifications. RNA-seq data have already provided promising anatomical information, with some of these populations corresponding particularly well to the previously described neurochemical classification. For example, among inhibitory interneurons, the GAL/DYN + , NPY + , CR + and PV + populations identified in laminae I–II seem to include cells in the GABA clusters 1–3, 5–7, 8–9 and 14 of Haring et al. (2018), respectively (Fig. 2). Among excitatory neurons in this region, the CCK + , NTS + , NKB + , NPFF + and SP + cells include those in the GLUT clusters 2–3, 4, 5–7, 9 and 10–11 of Haring et al. (2018), respectively (Fig. 2). Most importantly, the transcriptomic data also reveal the cellular content of DH neuronal populations and already confirms previous observations, for example identifying neurons of the DE-4 cluster of Sathyamurthy et al (2018) that express PKCγ, but not the delta opioid receptor (DOR) (Wang et al. 2018). However, the clusters defined by these studies are often extensive in their laminar spread (Haring et al. 2018). For example the Glut10–11 clusters, which include excitatory SP + cells, extend throughout all DH laminae. Since Dickie et al. (2018) identified a distinctive population of SP + neurons in lamina II, it will be important to determine the extent to which the transcriptomic clusters contain functionally distinct subsets of DH neurons, and whether this fits current knowledge of DH neuronal populations (Koch et al. 2018). Functional analysis of DH neurons in pain processing has also begun using transcriptomic data, by combining detection of the immediate-early gene Arc or cFos, with markers of identified cell types using immunohistochemistry or in situ hybridization multiplexing, in pathophysiological conditions (Haring et al. 2018; Sathyamurthy et al. 2018).

Most importantly, these transcriptomic analyses provide putative markers for cell clusters located in the superficial, but also deep DH, and may reveal the as-yet unknown chemical phenotype of several superficial neurons and most of deep DH neurons. Additionally, it has recently been shown that ion channel density distributions, and particularly densities of K_V_1- and A-type potassium conductances, can predict neuronal firing pattern in the DH (Balachandar and Prescott 2018). It will be thus very interesting to see if transcriptomic expression of potassium conductance-related channels from the newly identified cell clusters can predict the firing pattern properties of previously recorded DH neurons, in physiological conditions but also during persistent pain states.

Of note however, future investigations will have to test whether this new classification of DH neurons can be applied to different dermatomes and to different species, including humans. For example, in mice, expression of the neuronal calcium-binding protein NECAB1 is used to label the cluster DE-5 in Sathyamurthy et al. (2018), and is strongly expressed in the Glut5 cluster in Haring et al. (2018). However, NECAB1 is expressed almost exclusively by excitatory neurons in the superficial laminae, excluding the PKCγ + population, in mice, but by half of inhibitory neurons expressing the SOM receptor SST2R and half of excitatory PKCγ + neurons in rats (Zhang et al. 2014, 2016). On the other hand, NECAB2 labels similar populations of neurons in the inner part of lamina II in mice, rats and humans (Zhang et al. 2014, 2016), but is expressed by several clusters defined by Haring et al. (2018) and Sathyamurthy et al. (2018). There are also important differences in gene expression in distinct spinal segments and DH territories innervated by hairy and glabrous skin, which may be missed in current transcriptomic clustering of DH neurons. Clusters of Haring et al. (2018), and most likely Sathyamurthy et al. (2018) as well, are widely distributed in the medio-lateral axis and were defined by cells originating from several somatotopic territories. For example, excitatory DYN + neurons are included in the DE-15 (Sathyamurthy et al. 2018) and Glut14 (Haring et al. 2018) clusters. However, excitatory DYN + neurons are largely restricted to mid-lumbar DH (corresponding to glabrous skin territory) (Huang et al. 2018), and transcriptomic clusters do not seem to reflect these differences. Finally, it will be critical to determine whether these molecular markers remain unchanged after injury, as mRNA expression undergoes important regulations in persistent pain states (Tansley et al. 2018; Uttam et al. 2018).

## Current advances in dorsal horn neurons associated with cutaneous mechanical allodynia

### Cutaneous mechanical allodynia

CMA is a highly debilitating symptom of chronic pain reported by about 45% of patients, for whom normally innocuous stimulation of the skin is perceived as painful (Bouhassira et al. 2005). A similar prevalence of CMA is found in patients with pain syndromes that are associated with nervous system or somatic lesions, including nerve trauma or inflammatory arthropathies (Bouhassira et al. 2005). CMA can be generated following a gentle movement (dynamic CMA) or a blunt pressure (static CMA) applied to the surface of the skin. Prevalence of static CMA does not differ between patients with or without neuropathic characteristics, whereas dynamic CMA preferentially affects patients with neuropathic pain (Bouhassira et al. 2005). The large range of terms (burning, stabbing, electric shocks…) and conditions associated with this disease illustrates well that CMA is multidimensional and related to highly heterogeneous symptoms, mechanisms and neural circuits (Bouhassira et al. 2008; Peirs and Seal 2016).

### The dorsal horn: a critical entry point for cutaneous mechanical allodynia

Understanding of chronic pain circuits, including those responsible for CMA, is currently limited, but yet critical for the development of an efficient evidence-based therapeutic approach (Freeman et al. 2014). Nevertheless, neuronal circuits underlying CMA have been the subject of numerous investigations, with a large contribution from preclinical animal studies (Braz et al. 2014; Lechner 2017; Peirs and Seal 2016). During the last decade, there has been particular interest in understanding CMA neural circuits within the DH. Indeed, selective loss of a large proportion of DH neurons, through conditional deletion of the testicular orphan nuclear receptor 4, results in near complete absence of supraspinal integrated pain behaviors (Wang et al. 2013), revealing the critical role of the DH in chronic pain processing. Frustratingly however, an accurate description of DH neuronal circuits engaged during CMA remains difficult to achieve. Part of the reason for this relates to the fact that many studies have investigated relatively large neuronal populations that may be somewhat heterogeneous. Thus, it is often difficult to identify which subpopulation may actually participate in CMA, where within the DH and how. Furthermore, evidence suggests that the phenotype of DH neurons engaged during CMA is closely correlated with the pain etiology, changing our view of the neuronal circuitry to specific microcircuits underlying the symptom.

### Dorsal horn neurons associated with persistent cutaneous mechanical allodynia

#### Persistent neuropathic pain

Neuropathic pain affects 7–10% of the general population and results from a lesion affecting peripheral or central neurons (Colloca et al. 2017). Etiology for chronic neuropathic pain is multifactorial, and this has led to the development of several animal models (Coderre and Laferriere 2019; Kumar et al. 2018), potentially associated with different neuronal circuits. Neuronal populations engaged in the DH during neuropathic pain were historically revealed using immunohistochemistry directed against immediate-early genes (a.k.a. primary response genes) such as cFos, on DH slices. Analysis of these so-called “activity markers” in the DH after sciatic nerve lesion reveals cells located in the most superficial DH, through the injured segment ipsilateral to the nerve lesion, but also some scattered cells in deeper laminae from III to V. Interestingly, the distribution of cFos + cells within the DH changes over time, with numerous cells located in laminae I–II immediately after the nerve lesion, but which spread to deeper laminae from 2 days to 4 weeks after the injury (Chi et al. 1993).

In is important to note that prolonged general anesthesia may affect the expression of these activity markers. Indeed, the DH neuronal activity of non-anesthetized animals includes ongoing activity related to the nerve trauma itself, but also activity induced by mechanical, and sometimes chemical or thermal, unpredicted stimulations of the limbs and body that are invariably generated by the animal motor behaviors. Thus, in deeply anesthetized neuropathic mice (e.g., in the absence of peripheral stimulus), expression of the activity marker pERK1/2 or cFos is only slightly increased in superficial DH compared to sham animals (Liu et al. 2018; Peirs et al. 2015), suggesting that DH neuronal plasticity, rather than ongoing activity, remains for an extended period of time after nerve lesion. Importantly, such plastic changes seem to occur only in the injured, but not adjacent, segment in rats (Lu et al. 2013). The molecular identity of these activated cells is currently unknown.

##### Neuropathic static cutaneous mechanical allodynia

As mentioned above, peripheral neuropathy can induce static and/or dynamic CMA in 46% and 41% of patients, respectively (Bouhassira et al. 2005). The facts that: (1) disruption of DH GABAergic (Peirs et al. 2016) or glycinergic (Miraucourt et al. 2009) inhibition induces static or dynamic CMA, respectively, (2) morphine dose dependently blocks static but not dynamic CMA, and (3) static and dynamic CMA seem to be preferentially signaled by Aδ and Aβ afferents, respectively (Field et al. 1999), suggest that different DH circuits underlie these symptoms.

In anesthetized neuropathic mice, static mechanical stimulation of the skin using innocuous von Frey filaments recruits cells in the DH that extend from lamina I to VI (Peirs et al. 2015). Using cFos as an activity marker, it was reported that about 70% of DH neurons activated during neuropathic static CMA are PAX2-negative, suggesting that about two-thirds of this neuronal circuit is excitatory (Peirs et al. 2015). Co-immunolabeling of cFos with neuronal markers previously described reveals activity in excitatory neurons that transiently express VGLUT3 in lamina III, and PKCγ and CR neurons in lamina II. These cells account for 24% of all cFos + cells in laminae I–III. Because in these conditions, 29% of laminae I–III cFos + cells are inhibitory, we can estimate that VGLUT3 + , PKCγ + and CR + neurons account for 34% of all excitatory neurons expressing cFos during neuropathic static CMA in this area. Of note, selective intersectional ablation of DH VGLUT3 + or CR + neurons does not affect neuropathic static CMA (Cheng et al. 2017; Duan et al. 2014), suggesting that these cells are engaged but not required in these conditions. Selective inhibition of DH PKCγ + neurons in neuropathic conditions has not been described yet. Nonetheless, pharmacological inhibition (Petitjean et al. 2015) or virally mediated down-regulation (Zou et al. 2011) of the DH PKCγ kinase strongly reduces static mechanical allodynia in rats and mice after sciatic nerve injury, suggesting that this population is necessary for the expression of neuropathic static CMA.

Other excitatory DH neurons also participate in neuropathic static CMA. Selective intersectional ablation of DH neurons expressing SOM significantly reduces static CMA following sciatic nerve injury (Duan et al. 2014). However, SOM is expressed by many excitatory neurons in laminae I–II, including most of those belonging to each of the six neurochemically defined populations shown in Fig. 2, suggesting that this ablation strategy is likely to have affected a large proportion of superficial DH excitatory interneurons belonging to different functional populations (Gutierrez-Mecinas et al. 2019a; Todd 2017). Selective ablation of DH excitatory neurons expressing the NPY receptor NPYY1R, using intrathecal delivery of NPY-saporin, significantly reduces neuropathic static CMA in rats (Nelson et al. 2019). Interestingly, such intervention also reduces cold hypersensitivity induced by nerve injury, which is reported by 28% of patients with neuropathic pain (Bouhassira et al. 2005). However, it is unclear which of the different types of neurons that have been ablated is responsible for the behavioral effect, as NPYY1R is expressed by numerous excitatory neurons in the superficial DH including SOM + and CR + (but not PKCγ +) neurons (Nelson et al. 2019). Similarly, virally mediated ablation of the large population of DH CCK + cells nearly abolishes static CMA after peripheral nerve injury in mice (Liu et al. 2018). Saporin-mediated ablation of DH NK1R + neurons also strongly reduces neuropathic static CMA after ligation of the L5 and L6 spinal nerves in rats (Nichols et al. 1999). Because 80% of projection neurons in lamina I express NK1R in rats, this major spinal output of the ALT is likely to have been substantially affected by the saporin treatment. However, as indicated earlier, other cells mostly located in laminae III and IV also express NK1R, and may be included among those ablated with substance P-saporin. It is thus unclear which of the NK1R + cells are responsible for the behavioral effect observed after cellular ablation. Of note, selective intersectional ablation of TAC2 + DH neurons (i.e., those that express neurokinin B) did not affect neuropathic CMA in mice (Duan et al. 2014).

It is commonly accepted that chronic neuropathic pain is associated with reduced neuronal inhibition in the DH (Gradwell et al. 2019; Zeilhofer et al. 2012a), and that restoring spinal inhibition, using GABAergic cell transplants for example (Braz et al. 2012), has a therapeutic potential to relieve neuropathic CMA. Accordingly, selective activation of DH PV + (Petitjean et al. 2015) or GLYT2 + (Foster et al. 2015) inhibitory neurons, using virally delivered designer receptors exclusively activated by designer drugs (DREADDs), strongly reduces static CMA following sciatic nerve injury in mice. Similarly, selective activation of deep DH inhibitory interneurons that express RET, using virally delivered excitatory DREADDs, significantly reduces static CMA induced by L4 spinal nerve ligation in mice, whereas ablation of the cells enhances it even more (Cui et al. 2016). In contrast, selective intersectional ablation of DH neurons expressing DYN does not aggravate static CMA after spared nerve injury in mice (Duan et al. 2014). However, it is important to note that DYN + ablated mice spontaneously develop static CMA, and it is thus possible that nerve injury affects these cells, leading to neuropathic static CMA. Similarly, selective ablation of DH PV + inhibitory neurons using virally delivered saporin leads to static CMA in mice, and as indicated above, DH PV + neurons are involved in neuropathic static CMA (Petitjean et al. 2015).

##### Neuropathic dynamic cutaneous mechanical allodynia

Less is known about DH circuits underlying neuropathic dynamic CMA. In anesthetized mice with nerve injury, innocuous stimulation of the skin with a paintbrush evokes intense activity in the whole DH, including in neurons expressing CCK in laminae III–IV and NK1R in lamina I (Liu et al. 2018). As for static CMA, virally mediated ablation of DH CCK + cells nearly abolishes dynamic CMA after peripheral nerve injury in mice (Liu et al. 2018). Interestingly, selective intersectional ablation of DH VGLUT3 + neurons also strongly reduces neuropathic dynamic CMA in mice (Cheng et al. 2017), indicating that while these cells are dispensable for static neuropathic CMA, they are required for the dynamic form of neuropathic CMA. Selective intersectional ablation of DH neurons expressing SOM significantly reduces dynamic CMA following sciatic nerve injury in mice (Duan et al. 2014) but, as indicated above, it is unclear which subpopulation(s) of SOM + cells is/are responsible for this effect. As for neuropathic static CMA, selective intersectional ablation of DH CR + neurons does not affect dynamic CMA after sciatic nerve injury, indicating that CR neurons are not required for any form of CMA in neuropathic conditions (Duan et al. 2014).

Very little is known about DH inhibitory neurons that participate in neuropathic dynamic CMA. Similar to neuropathic static CMA, selective intersectional ablation of DH neurons expressing DYN does not affect dynamic CMA after spared nerve injury in mice (Duan et al. 2014). However, it is important to note that DYN + ablated mice also spontaneously develop dynamic CMA, and activity from these cells could be affected by nerve lesions, leading to neuropathic dynamic CMA. Similarly, selective inhibition or ablation of DH PV + inhibitory neurons using respectively virally delivered tetanus toxin light chain or saporin, leads to dynamic CMA and cFos activity in the DH induced by brush stimulation in mice, suggesting that DH PV + neurons might also be involved in neuropathic dynamic CMA (Boyle et al. 2019; Petitjean et al. 2015).

#### Persistent inflammatory pain

Inflammatory pain can be associated with a number of diseases and results from the local release of inflammatory signaling molecules from immune cells, including cytokines, growth factors and prostaglandins. Symptoms associated with inflammatory pain include redness, warmth and swelling of the affected area, but also manifestations that can overlap with those of neuropathic pain, such as CMA (Vardeh et al. 2016). Importantly, inflammatory mediators such as nitric oxide and tumor necrosis factor-α (TNF-α) can induce nerve damage and also elements of neuropathic pain. There are thus several common mechanisms associated with neuropathic and inflammatory pain (Xu and Yaksh 2011), but also major differences, such as pharmacological responsiveness to non-steroidal anti-inflammatory drugs (NSAIDs) and opiates (Lynch and Watson 2006), suggesting that mechanisms, and by extension neuronal circuits engaged during CMA associated with inflammatory pain, might differ from those associated with neuropathic CMA.

Several animal models have been developed to study specific pathologies associated with inflammatory pain, such as multiple sclerosis (MS) (Procaccini et al. 2015) or migraine (Chou and Chen 2018; Dallel et al. 2018), but most common models of inflammatory pain involve injections of agents such as capsaicin, formalin, mustard oil, carrageenan or complete Freund’s adjuvant into the skin, muscle, visceral organs or joints (Coderre and Laferriere 2019; Gregory et al. 2013). Virtually, all of these models are associated with immediate CMA, with some lasting longer due to chronic inflammation, such as the carrageenan and CFA pain models for which CMA can persist from days to several weeks. In rats, CFA-induced inflammation rapidly increases cFos and pERK expression in the DH, predominantly in laminae I–II, and these remain upregulated for at least 24–48 h following the injection (Geranton et al. 2008; Ji et al. 2002). Neurons that are active 48 h after CFA injection include lamina I NK1R + and DYN + neurons in the superficial DH, but also a few DYN + neurons of the deep DH (Ji et al. 2002). However, and as for nerve injury, results from these studies were obtained from behaving animals and may include cells directly activated by the injury, but also cells activated by stimulation of the skin due to motor behavior. Indeed, the DH of anesthetized mice that had undergone prolonged anesthesia 24 h after carrageenan or CFA treatment (i.e. in the absence of peripheral stimulus) displays very scattered cells through laminae I–VI, suggesting again that DH neuronal plasticity, rather than ongoing activity, remains for an extended period of time following long-term tissue inflammation (Gao and Ji 2010; Peirs et al. 2015).

##### Inflammatory static cutaneous mechanical allodynia

In anesthetized mice with carrageenan-induced persistent inflammation, static mechanical stimulation of the skin with innocuous von Frey filaments elicits cFos in cells located predominantly in the medial part of the DH lamina I and II, but also through the deeper layers, mostly in laminae IV–VI (Peirs et al. 2015). In another study, innocuous static stimulation of the skin (by a cotton tip) of anesthetized rats treated 24 h earlier with CFA, increased pERK + neurons in the DH lamina I and the outer part of lamina II (Gao and Ji 2010). Interestingly, 59% of cells activated by inflammatory static CMA do not express the inhibitory marker PAX2, suggesting that fewer excitatory neurons, or more inhibitory neurons, are engaged during inflammatory static CMA compared to CMA in neuropathic conditions (Peirs et al. 2015). Co-immunolabeling of cFos with neuronal markers reveals activity in excitatory neurons that transiently express VGLUT3 in lamina III, and CR neurons in lamina II in mice (Peirs et al. 2015). These cells account for a third of cFos + cells in laminae I–III, which we can estimate to be about 56% of all excitatory neurons expressing cFos during inflammatory static CMA in this area. Similar to neuropathic injury, selective intersectional ablation of VGLUT3 + neurons does not alter inflammatory static CMA induced by subcutaneous injection of CFA in mice (Cheng et al. 2017), suggesting again that VGLUT3 + neurons are engaged in, but not required for, any form of static CMA. Interestingly, neither pERK1/2 nor cFos has been reported in DH excitatory neurons expressing PKCγ during static CMA in persistent inflammatory pain models in either rats or mice (Gao and Ji 2010; Peirs et al. 2015). Of note, however, activation of lamina II PKCγ + neurons has been observed rapidly following static innocuous stimulation of the skin in rats treated 70 min before with CFA, suggesting that CMA associated with immediate, but not persistent, inflammation involves PKCγ + cells (Alba-Delgado et al. 2018).

Selective intersectional ablation (Duan et al. 2014) or inhibition (Christensen et al. 2016) of DH excitatory SOM + neurons, which, as noted before, include several neurochemically defined populations, results in a dramatic reduction of inflammatory static CMA induced by CFA injection in mice. As mentioned above, neuronal activity has been reported in lamina I NK1R + neurons in behaving animals treated with CFA, suggesting that these cells may also participate in the DH circuit underlying inflammatory static CMA (Ji et al. 2002). Accordingly, saporin-mediated ablation of DH NK1R + neurons significantly reduces static CMA induced by a subcutaneous injection of carrageenan or CFA in rats (Nichols et al. 1999). As for neuropathic CMA, it will be interesting to identify which subpopulation of NK1R + cells is responsible for this behavioral effect.

Interestingly, in contrast to neuropathic conditions, increasing spinal inhibition using GABAergic cell transplants does not affect inflammatory static CMA in mice (Braz et al. 2012), suggesting a different role of DH inhibition in neuropathic versus inflammatory CMA. Nevertheless, similar to neuropathic static CMA, virally mediated ablation of inhibitory RET + neurons enhances static CMA induced by CFA injection in mice (Cui et al. 2016). In contrast, selective intersectional ablation of DH inhibitory DYN + (Duan et al. 2014) or NPY + (Bourane et al. 2015a) neurons does not affect static CMA following injection of CFA in mice. However, static CMA spontaneously develops in DYN + , but not NPY + , ablated animals, and DYN + neurons might be affected during inflammation, leading to inflammatory static CMA. In contrast to neuropathic pain, the effect of manipulating DH inhibitory neurons expressing PV, NKB or GLYT2 has not been reported for inflammatory static CMA.

##### Inflammatory dynamic cutaneous mechanical allodynia

Almost nothing is known about DH neuronal circuits underlying inflammatory dynamic CMA. Part of the reasons for this surprising gap is the fact that dynamic CMA is very rarely reported by patients without neuropathy (Bouhassira et al. 2005), and does not always develop in animal models of persistent inflammatory pain (Peirs, unpublished observation). Nevertheless, selective intersectional ablation of DH excitatory SOM + (Duan et al. 2014) or VGLUT3 + (Cheng et al. 2017) neurons strongly reduces inflammatory dynamic CMA induced by CFA injection in mice, indicating that these cells are critical for all forms of dynamic CMA.

Ablation of DH inhibitory DYN + neurons does not affect dynamic CMA following injection of CFA in mice (Duan et al. 2014), but once again DYN + ablated mice spontaneously develop dynamic CMA, which makes it difficult to be sure about the role of these cells in inflammatory dynamic CMA.

### Understanding cutaneous mechanical allodynia circuitry: pitfalls and future directions

While there has been considerable progress in revealing the circuitry for CMA, several potential pitfalls must be taken into consideration. For example, genetic strategies aimed at selectively targeting populations of cells do not always distinguish “transiently expressing” populations from those that continue to express the targeted gene, and this is likely to be a particular issue when targeting peptide-expressing populations. In contrast, tamoxifen-inducible Cre lines, or local injections of viral vectors directly into the DH, only target neurons in the adult but often capture only a fraction of the global cell population (Abraira et al. 2017; Gutierrez-Mecinas et al. 2019b; Peirs et al. 2015).

In the last few years, intersectional genetic strategies have been used to dissect out the DH circuitry, by using mouse lines, in which transgene expression depends on both Cre-lox and FlpO-FRT recombinations. Targeting of selective DH neurons has been achieved using conditional expression of Cre or FlpO, driven by either Cdx2 or Lbx1 promoters that are expressed only in neurons derived from the dorsal spinal cord and the dorsal hindbrain (Bourane et al. 2015b; Duan et al. 2014). Double conditional expression typically occurs in about 85% of the entire targeted DH population, depending on the proportion of DH neurons that express the two conditional genes. While these studies are already groundbreaking, future investigations may need to involve even more complex intersections of genes, to distinguish more discrete subpopulations of cells, for example excitatory versus inhibitory cells, or projection neurons versus interneurons, where these co-exist within the currently defined populations (Abraira et al. 2017; Haring et al. 2018; Sathyamurthy et al. 2018). Indeed, a recent study identified DH neurons expressing TAC1 (the gene coding for SP) located mainly in the superficial layers that specifically transmit the aversive component of pain, without affecting reflex nocifensive responses (Huang et al. 2019). The strategy used to ablate SP + neurons in this study presumably captured both interneurons and lamina I ALT projection neurons, making it difficult to distinguish their relative contributions. In addition, a minority of SP + cells are inhibitory (Gutierrez-Mecinas et al. 2017) and ablating these cells may have contributed to the behavior. Finally, some excitatory interneurons (including many of the PKCγ + cells in laminae II–III) appear to express SP during development (Gutierrez-Mecinas et al. 2017) and may have been included among those that were ablated in this study. Importantly however, regardless of which subpopulation of SP + cells that was affected, the study does point to a potential role of DH populations in CMA that may have been overlooked in the previous studies described above, which used reflex behavior as a readout of pain, since these nocifensive responses can be independent of the cognitive and emotional aspects of pain.

## Conclusion

**Fig. 3:**
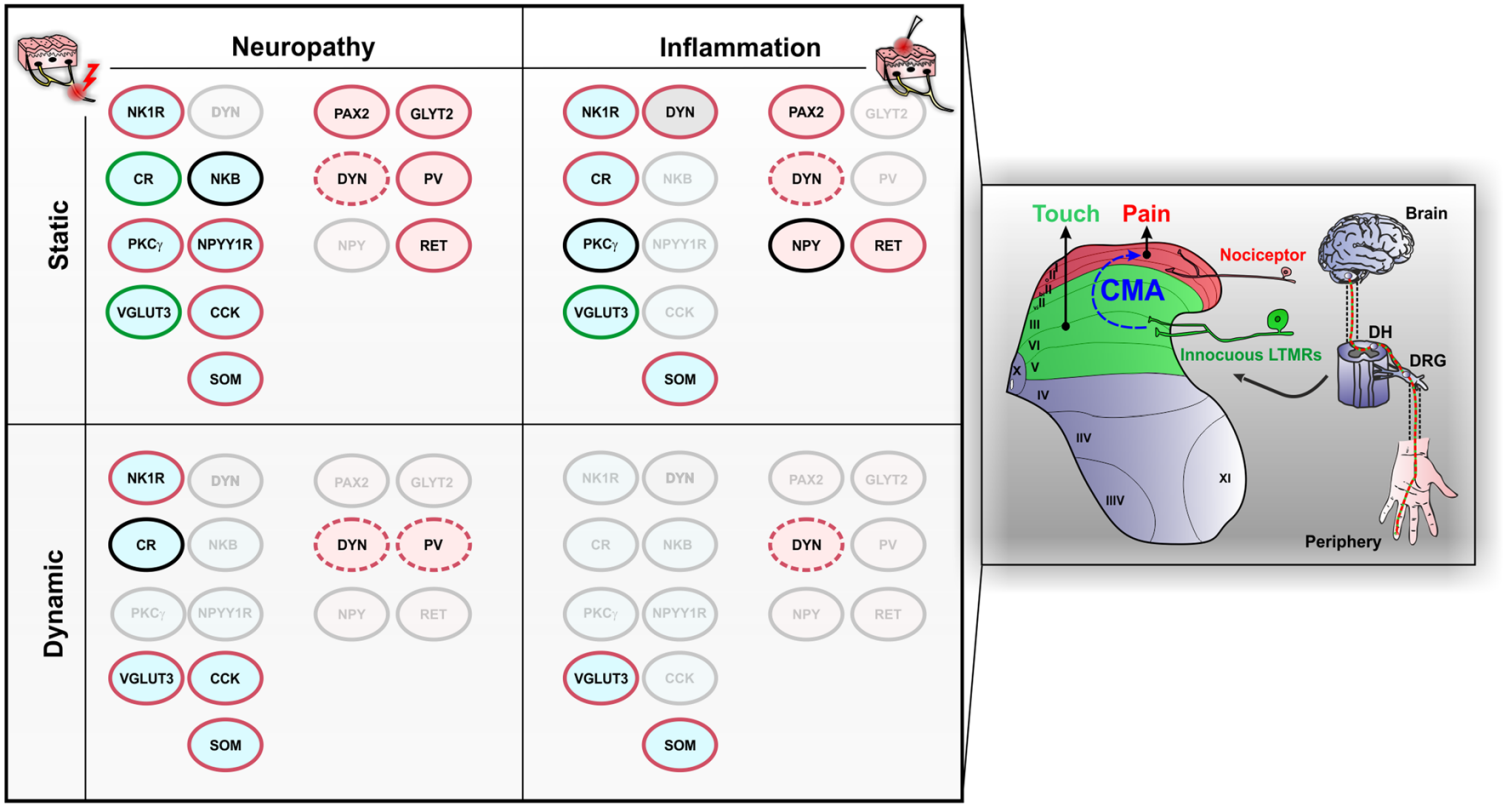
Populations of dorsal horn neurons involved in cutaneous mechanical allodynia. Schematic representation on the right illustrates the segregation of touch and pain in the spinal cord dorsal horn. In physiological conditions, non-nociceptive sensory neurons (LTMRs) innervate the deep DH (green), whereas nociceptive fibers terminate in the superficial laminae (red). During cutaneous mechanical allodynia (CMA), touch induces pain through activation of a polysynaptic circuit (blue) that connects the non-nociceptive deep DH to the pain‐related superficial layers. Populations of neurons that have been implicated in CMA are indicated on the left part of the figure, with distinctions between static/dynamic and neuropathic/inflammatory CMA. Populations of neurons that are mostly excitatory are displayed in light blue ellipses and those that are mostly inhibitory neurons are displayed in light pink ellipses. Lamina I neurons expressing dynorphin (DYN) include both inhibitory and excitatory cells and are displayed as gray ellipses. Populations of neurons that have not been tested for the respective conditions are transparent. Outlines in red indicate that the population has been implicated in the expression of CMA, in green indicates that the population is engaged but not required for CMA and in black indicates that the population is dispensable for CMA. Dotted outlines indicate that the requirement of the population for CMA is unclear. *DH* dorsal horn, *DRG* dorsal root ganglion, *LTMR* low‐threshold mechanoreceptor, *NK1R* neurokinin 1 receptor, *CR* calretinin, *PKCγ* gamma isoform of protein kinase C, *VGLUT3* vesicular glutamate transporter 3, *DYN* dynorphin, *NKB* neurokinin B, *NPYY1R* neuropeptide Y Y1 receptor, *CCK* cholecystokinin, *SOM* somatostatin, *PAX2* paired box gene 2, *NPY* neuropeptide Y, *GLYT2* glycine transporter 2, *PV* parvalbumin, *RET* receptor tyrosine kinase

The DH is a critical entry point in the central nervous system, responsible for integrating and modulating complex multidimensional sensory information. Recent advances in genetic tools and mass single cell analysis have revealed the remarkable heterogeneity of DH neurons and CMA circuitry. Importantly, the improving picture of DH composition reveals multiple circuits for chronic pain, including those dedicated to a unique symptom such as CMA (Fig. 3). Such multiplicity and apparent redundancy in pain circuits most likely relate to the high variability in responsiveness of chronic pain patients to therapeutic intervention, thus stressing the need for a careful consideration of symptomatology, pain etiology and neuronal circuitry, in both preclinical investigations and clinical trials (Attal et al. 2011; Dallel and Voisin 2001).

As we move forward, several challenges will require particular attention. An important unanswered question is where the circuit underlying CMA is initiated within the DH. Historical views of the DH circuitry for CMA drew a dorsally directed polysynaptic circuit originating around lamina II, which eventually engages nociceptive ALT neurons in lamina I. Several entry points have now been proposed, such as lamina III VGLUT3 + neurons (Peirs et al. 2015), neurons in lamina II_i_ that express PKCγ (Miraucourt et al. 2007) or CR (Petitjean et al. 2019), or vertical cells in lamina II_o_ (Boyle et al. 2019). In fact, tactile stimulation of the skin, which ultimately engages CMA circuits, activates a large range of both myelinated and unmyelinated primary afferents, with various conduction velocities and central terminal expression patterns within the DH (Abraira and Ginty 2013). Tactile information is thus transmitted to various locations in the DH at very specific times and action potential firing properties of DH neurons are likely to play a critical role in the temporal transmission of the signal to projection neurons, with coded amplitude, frequency and rhythm. Understanding of such complex timing mechanisms of DH modulation and integration is often overlooked, but will be critical for the deciphering of the DH circuitry of CMA (Pagani et al. 2019; Zhang et al. 2018).

Second, we still have poor understanding of the DH neuronal connectivity, including inputs from primary sensory neurons and interconnectivity of DH interneurons (Cordero-Erausquin et al. 2016; Kato et al. 2009). Importantly, recently developed powerful tools are now available to dissect out the DH neuronal connectivity associated with molecularly defined neurons, with a remarkable level of detail. For example, numerous cells can now be labeled within a single DH slice using viruses or genetic alleles carrying the sequences of several fluorescent proteins, such that recombination leads to random expression of one or more of these proteins (Cai et al. 2013). Use of such genetic tools results in a wide range of hues in the same tissue and has been used to reconstruct the dendritic arbors and terminal axons of numerous SP + cells in the DH (Dickie et al. 2018). Several studies have also used retrograde transynaptic tracing to identify neurons that are presynaptic to genetically labeled cells (Wickersham et al. 2007). This powerful technique has been successfully used to characterize the molecular phenotype of primary afferent and supraspinal descending inputs that target excitatory DH neurons expressing RORα (Bourane et al. 2015b), GRP (Sun et al. 2017), ZIC2 (Paixão et al. 2019), and inhibitory glycinergic (Foster et al. 2015), enkephalinergic and GABAergic (Francois et al. 2017) DH neurons. Surprisingly however, retrograde transynaptic tools have not been used to identify intraspinal connectivity. In contrast, anterograde transneuronal tracing of DH Cre-expressing cell lines, using cre-dependent viruses expressing wheat germ agglutinin (WGA), has led to the identification of several postsynaptic targets of excitatory VGLUT3 + neurons (Peirs et al. 2015) and inhibitory neurons expressing the vesicular GABA transporter (VGAT) (Francois et al. 2017) within the DH. Comparing the synaptic connectivity to the function of DH neuronal populations will be critical for drawing the long awaited functional map of the DH circuitry for CMA.

## Electronic supplementary material

Below is the link to the electronic supplementary material.

Supplementary file1 (PDF 1834 kb)

## References

[CR1] Abraira VE, Ginty DD (2013). The sensory neurons of touch. Neuron.

[CR2] Abraira VE (2017). The cellular and synaptic architecture of the mechanosensory dorsal horn. Cell.

[CR3] Al Ghamdi KS, Polgar E, Todd AJ (2009). Soma size distinguishes projection neurons from neurokinin 1 receptor-expressing interneurons in lamina I of the rat lumbar spinal dorsal horn. Neuroscience.

[CR4] Al Khater KM, Kerr R, Todd AJ (2008). A quantitative study of spinothalamic neurons in laminae I, III, and IV in lumbar and cervical segments of the rat spinal cord. J Comp Neurol.

[CR5] Alba-Delgado C, El Khoueiry C, Peirs C, Dallel R, Artola A, Antri M (2015). Subpopulations of PKCγ interneurons within the medullary dorsal horn revealed by electrophysiologic and morphologic approach. Pain.

[CR6] Alba-Delgado C, Mountadem S, Mermet-Joret N, Monconduit L, Dallel R, Artola A, Antri M (2018). 5-HT2A receptor-induced morphological reorganization of PKCγ-expressing interneurons gates inflammatory mechanical allodynia in rat. J Neurosci.

[CR7] Albisetti GW (2019). Dorsal horn gastrin-releasing peptide expressing neurons transmit spinal itch but not pain signals. J Neurosci.

[CR8] Al-Khater KM, Todd AJ (2009). Collateral projections of neurons in laminae I, III, and IV of rat spinal cord to thalamus, periaqueductal gray matter, and lateral parabrachial area. J Comp Neurol.

[CR9] Almarestani L, Waters SM, Krause JE, Bennett GJ, Ribeiro-da-Silva A (2007). Morphological characterization of spinal cord dorsal horn lamina I neurons projecting to the parabrachial nucleus in the rat. J Comp Neurol.

[CR10] Andrew D, Craig AD (2001). Spinothalamic lamina I neurons selectively sensitive to histamine: a central neural pathway for itch. Nat Neurosci.

[CR200] Artola A, Voisin D, Dallel R (2020) PKCγ interneurons, a gateway to pathological pain in the dorsal horn. J Neural Transm (Vienna). 10.1007/s00702-020-02162-6 10.1007/s00702-020-02162-6 32108249

[CR12] Attal N (2011). Assessing symptom profiles in neuropathic pain clinical trials: can it improve outcome?. Eur J Pain.

[CR13] Balachandar A, Prescott SA (2018). Origin of heterogeneous spiking patterns from continuously distributed ion channel densities: a computational study in spinal dorsal horn neurons. J Physiol.

[CR15] Baseer N, Polgar E, Watanabe M, Furuta T, Kaneko T, Todd AJ (2012). Projection neurons in lamina III of the rat spinal cord are selectively innervated by local dynorphin-containing excitatory neurons. J Neurosci.

[CR16] Bice TN, Beal JA (1997). Quantitative and neurogenic analysis of neurons with supraspinal projections in the superficial dorsal horn of the rat lumbar spinal cord. J Comp Neurol.

[CR17] Bice TN, Beal JA (1997). Quantitative and neurogenic analysis of the total population and subpopulations of neurons defined by axon projection in the superficial dorsal horn of the rat lumbar spinal cord. J Comp Neurol.

[CR18] Bouhassira D (2005). Comparison of pain syndromes associated with nervous or somatic lesions and development of a new neuropathic pain diagnostic questionnaire (DN4). Pain.

[CR19] Bouhassira D, Lanteri-Minet M, Attal N, Laurent B, Touboul C (2008). Prevalence of chronic pain with neuropathic characteristics in the general population. Pain.

[CR20] Bourane S (2015). Gate control of mechanical itch by a subpopulation of spinal cord interneurons. Science.

[CR21] Bourane S (2015). Identification of a spinal circuit for light touch and fine motor control. Cell.

[CR22] Boyle KA (2017). A quantitative study of neurochemically defined populations of inhibitory interneurons in the superficial dorsal horn of the mouse spinal cord. Neuroscience.

[CR23] Boyle KA (2019). Defining a spinal microcircuit that gates myelinated afferent input: implications for tactile allodynia. Cell Rep.

[CR24] Braz JM, Sharif-Naeini R, Vogt D, Kriegstein A, Alvarez-Buylla A, Rubenstein JL, Basbaum AI (2012). Forebrain GABAergic neuron precursors integrate into adult spinal cord and reduce injury-induced neuropathic pain. Neuron.

[CR25] Braz J, Solorzano C, Wang X, Basbaum AI (2014). Transmitting pain and itch messages: a contemporary view of the spinal cord circuits that generate gate control. Neuron.

[CR26] Brown AG (1982). The dorsal horn of the spinal cord. Q J Exp Physiol.

[CR27] Brown AG, Fyffe RE (1981). Form and function of dorsal horn neurones with axons ascending the dorsal columns in cat. J Physiol.

[CR28] Brown JL, Liu H, Maggio JE, Vigna SR, Mantyh PW, Basbaum AI (1995). Morphological characterization of substance P receptor-immunoreactive neurons in the rat spinal cord and trigeminal nucleus caudalis. J Comp Neurol.

[CR29] Burstein R, Dado RJ, Giesler GJ (1990). The cells of origin of the spinothalamic tract of the rat: a quantitative reexamination. Brain Res.

[CR30] Cai D, Cohen KB, Luo T, Lichtman JW, Sanes JR (2013). Improved tools for the Brainbow toolbox. Nat Methods.

[CR31] Cameron D, Polgar E, Gutierrez-Mecinas M, Gomez-Lima M, Watanabe M, Todd AJ (2015). The organisation of spinoparabrachial neurons in the mouse. Pain.

[CR32] Cheng L (2017). Identification of spinal circuits involved in touch-evoked dynamic mechanical pain. Nat Neurosci.

[CR33] Chi SI, Levine JD, Basbaum AI (1993). Peripheral and central contributions to the persistent expression of spinal cord fos-like immunoreactivity produced by sciatic nerve transection in the rat. Brain Res.

[CR34] Chou TM, Chen SP (2018). Animal models of chronic migraine. Curr Pain Headache Rep.

[CR35] Christensen AJ (2016). In vivo interrogation of spinal mechanosensory circuits. Cell Rep.

[CR36] Coderre TJ, Laferriere A (2019). The emergence of animal models of chronic pain and logistical and methodological issues concerning their use. J Neural Transm (Vienna).

[CR37] Colloca L (2017). Neuropathic pain. Nat Rev Dis Primers.

[CR38] Cordero-Erausquin M, Allard S, Dolique T, Bachand K, Ribeiro-da-Silva A, De Koninck Y (2009). Dorsal horn neurons presynaptic to lamina I spinoparabrachial neurons revealed by transynaptic labeling. J Comp Neurol.

[CR39] Cordero-Erausquin M, Inquimbert P, Schlichter R, Hugel S (2016). Neuronal networks and nociceptive processing in the dorsal horn of the spinal cord. Neuroscience.

[CR40] Cortes R, Ceccatelli S, Schalling M, Hokfelt T (1990). Differential effects of intracerebroventricular colchicine administration on the expression of mRNAs for neuropeptides and neurotransmitter enzymes, with special emphasis on galanin: an in situ hybridization study. Synapse.

[CR41] Cui L (2016). Identification of early RET+ deep dorsal spinal cord interneurons in gating pain. Neuron.

[CR42] Dallel R, Voisin D (2001). Towards a pain treatment based on the identification of the pain-generating mechanisms?. Eur Neurol.

[CR43] Dallel R, Descheemaeker A, Luccarini P (2018). Recurrent administration of the nitric oxide donor, isosorbide dinitrate, induces a persistent cephalic cutaneous hypersensitivity: a model for migraine progression. Cephalalgia.

[CR44] Davidson S, Truong H, Giesler GJ (2010). Quantitative analysis of spinothalamic tract neurons in adult and developing mouse. J Comp Neurol.

[CR45] Del Barrio MG, Bourane S, Grossmann K, Schule R, Britsch S, O'Leary DD, Goulding M (2013). A transcription factor code defines nine sensory interneuron subtypes in the mechanosensory area of the spinal cord. PLoS ONE.

[CR46] Dickie AC (2018). Morphological and functional properties distinguish the substance P and gastrin-releasing peptide subsets of excitatory interneuron in the spinal cord dorsal horn. Pain.

[CR47] Ding YQ, Takada M, Shigemoto R, Mizumo N (1995). Spinoparabrachial tract neurons showing substance P receptor-like immunoreactivity in the lumbar spinal cord of the rat. Brain Res.

[CR48] Duan B (2014). Identification of spinal circuits transmitting and gating mechanical pain. Cell.

[CR49] Edgley SA, Gallimore CM (1988). The morphology and projections of dorsal horn spinocerebellar tract neurones in the cat. J Physiol.

[CR50] Fernandes EC, Santos IC, Kokai E, Luz LL, Szucs P, Safronov BV (2018). Low- and high-threshold primary afferent inputs to spinal lamina III antenna-type neurons. Pain.

[CR51] Field MJ, Bramwell S, Hughes J, Singh L (1999). Detection of static and dynamic components of mechanical allodynia in rat models of neuropathic pain: are they signalled by distinct primary sensory neurones?. Pain.

[CR52] Foster E (2015). Targeted ablation, silencing, and activation establish glycinergic dorsal horn neurons as key components of a spinal gate for pain and itch. Neuron.

[CR53] Francois A (2017). A brainstem-spinal cord inhibitory circuit for mechanical pain modulation by GABA and enkephalins. Neuron.

[CR54] Freeman R, Baron R, Bouhassira D, Cabrera J, Emir B (2014). Sensory profiles of patients with neuropathic pain based on the neuropathic pain symptoms and signs. Pain.

[CR56] Ganley RP (2015). Inhibitory interneurons that express GFP in the PrP-GFP mouse spinal cord are morphologically heterogeneous, innervated by several classes of primary afferent and include lamina I projection neurons among their postsynaptic targets. J Neurosci.

[CR57] Gao YJ, Ji RR (2010). Light touch induces ERK activation in superficial dorsal horn neurons after inflammation: involvement of spinal astrocytes and JNK signaling in touch-evoked central sensitization and mechanical allodynia. J Neurochem.

[CR58] Gatto G, Smith KM, Ross SE, Goulding M (2019). Neuronal diversity in the somatosensory system: bridging the gap between cell type and function. Curr Opin Neurobiol.

[CR59] Geranton SM, Fratto V, Tochiki KK, Hunt SP (2008). Descending serotonergic controls regulate inflammation-induced mechanical sensitivity and methyl-CpG-binding protein 2 phosphorylation in the rat superficial dorsal horn. Mol Pain.

[CR60] Gobel S (1975). Golgi studies in the substantia gelatinosa neurons in the spinal trigeminal nucleus. J Comp Neurol.

[CR61] Gobel S (1978). Golgi studies of the neurons in layer II of the dorsal horn of the medulla (trigeminal nucleus caudalis). J Comp Neurol.

[CR62] Gradwell MA, Callister RJ, Graham BA (2019). Reviewing the case for compromised spinal inhibition in neuropathic pain. J Neural Transm (Vienna).

[CR63] Gregory NS, Harris AL, Robinson CR, Dougherty PM, Fuchs PN, Sluka KA (2013). An overview of animal models of pain: disease models and outcome measures. J Pain.

[CR65] Grudt TJ, Perl ER (2002). Correlations between neuronal morphology and electrophysiological features in the rodent superficial dorsal horn. J Physiol.

[CR67] Gutierrez-Mecinas M, Furuta T, Watanabe M, Todd AJ (2016). A quantitative study of neurochemically defined excitatory interneuron populations in laminae I–III of the mouse spinal cord. Mol Pain.

[CR68] Gutierrez-Mecinas M (2017). Preprotachykinin A is expressed by a distinct population of excitatory neurons in the mouse superficial spinal dorsal horn including cells that respond to noxious and pruritic stimuli. Pain.

[CR69] Gutierrez-Mecinas M, Polgar E, Bell AM, Herau M, Todd AJ (2018). Substance P-expressing excitatory interneurons in the mouse superficial dorsal horn provide a propriospinal input to the lateral spinal nucleus. Brain Struct Funct.

[CR70] Gutierrez-Mecinas M, Bell A, Polgar E, Watanabe M, Todd AJ (2019). Expression of neuropeptide FF defines a population of excitatory interneurons in the superficial dorsal horn of the mouse spinal cord that respond to noxious and pruritic stimuli. Neuroscience.

[CR71] Gutierrez-Mecinas M, Bell AM, Shepherd F, Polgar E, Watanabe M, Furuta T, Todd AJ (2019). Expression of cholecystokinin by neurons in mouse spinal dorsal horn. J Comp Neurol.

[CR72] Gutierrez-Mecinas M (2019). Expression of calretinin among different neurochemical classes of interneuron in the superficial dorsal horn of the mouse spinal cord. Neuroscience.

[CR73] Hantman AW, Jessell TM (2010). Clarke’s column neurons as the focus of a corticospinal corollary circuit. Nat Neurosci.

[CR75] Haring M (2018). Neuronal atlas of the dorsal horn defines its architecture and links sensory input to transcriptional cell types. Nat Neurosci.

[CR76] Heinke B, Ruscheweyh R, Forsthuber L, Wunderbaldinger G, Sandkuhler J (2004). Physiological, neurochemical and morphological properties of a subgroup of GABAergic spinal lamina II neurones identified by expression of green fluorescent protein in mice. J Physiol.

[CR77] Huang J (2018). Circuit dissection of the role of somatostatin in itch and pain. Nat Neurosci.

[CR78] Huang T (2019). Identifying the pathways required for coping behaviours associated with sustained pain. Nature.

[CR79] Hughes DI, Sikander S, Kinnon CM, Boyle KA, Watanabe M, Callister RJ, Graham B (2012). Morphological, neurochemical and electrophysiological features of parvalbumin-expressing cells: a likely source of axo-axonic inputs in the mouse spinal dorsal horn. J Physiol.

[CR80] Iwagaki N, Garzillo F, Polgar E, Riddell JS, Todd AJ (2013). Neurochemical characterisation of lamina II inhibitory interneurons that express GFP in the PrP-GFP mouse. Mol Pain.

[CR81] Iwagaki N (2016). A combined electrophysiological and morphological study of neuropeptide Y-expressing inhibitory interneurons in the spinal dorsal horn of the mouse. Pain.

[CR82] Ji RR, Befort K, Brenner GJ, Woolf CJ (2002). ERK MAP kinase activation in superficial spinal cord neurons induces prodynorphin and NK-1 upregulation and contributes to persistent inflammatory pain hypersensitivity. J Neurosci.

[CR83] Kardon AP (2014). Dynorphin acts as a neuromodulator to inhibit itch in the dorsal horn of the spinal cord. Neuron.

[CR84] Kato G (2009). Organization of intralaminar and translaminar neuronal connectivity in the superficial spinal dorsal horn. J Neurosci.

[CR86] Kevetter GA, Haber LH, Yezierski RP, Chung JM, Martin RF, Willis WD (1982). Cells of origin of the spinoreticular tract in the monkey. J Comp Neurol.

[CR87] Koch SC (2017). RORbeta spinal interneurons gate sensory transmission during locomotion to secure a fluid walking gait. Neuron.

[CR88] Koch SC, Acton D, Goulding M (2018). Spinal circuits for touch, pain, and itch. Annu Rev Physiol.

[CR89] Kumar A, Kaur H, Singh A (2018). Neuropathic pain models caused by damage to central or peripheral nervous system. Pharmacol Rep.

[CR90] Lai HC, Seal RP, Johnson JE (2016). Making sense out of spinal cord somatosensory development. Development.

[CR91] Laing I, Todd AJ, Heizmann CW, Schmidt HH (1994). Subpopulations of GABAergic neurons in laminae I–III of rat spinal dorsal horn defined by coexistence with classical transmitters, peptides, nitric oxide synthase or parvalbumin. Neuroscience.

[CR92] Larsson M (2017). Pax2 is persistently expressed by GABAergic neurons throughout the adult rat dorsal horn. Neurosci Lett.

[CR93] Lechner SG (2017). An update on the spinal and peripheral pathways of pain signalling. e-Neuroforum.

[CR94] Levine AJ, Hinckley CA, Hilde KL, Driscoll SP, Poon TH, Montgomery JM, Pfaff SL (2014). Identification of a cellular node for motor control pathways. Nat Neurosci.

[CR95] Li JL, Li YQ, Kaneko T, Mizuno N (1999). Preprodynorphin-like immunoreactivity in medullary dorsal horn neurons projecting to the thalamic regions in the rat. Neurosci Lett.

[CR96] Li YQ, Li JL, Li H, Kaneko T, Mizuno N (2001). Protein kinase C gamma-like immunoreactivity of trigeminothalamic neurons in the medullary dorsal horn of the rat. Brain Res.

[CR97] Lima D, Coimbra A (1986). A Golgi study of the neuronal population of the marginal zone (lamina I) of the rat spinal cord. J Comp Neurol.

[CR98] Lima D, Avelino A, Coimbra A (1993). Morphological characterization of marginal (lamina I) neurons immunoreactive for substance P, enkephalin, dynorphin and γ-aminobutyric acid in the rat spinal cord. J Chem Neuroanat.

[CR100] Liu Y (2018). Touch and tactile neuropathic pain sensitivity are set by corticospinal projections. Nature.

[CR101] Lu Y, Perl ER (2005). Modular organization of excitatory circuits between neurons of the spinal superficial dorsal horn (laminae I and II). J Neurosci.

[CR102] Lu Y (2013). A feed-forward spinal cord glycinergic neural circuit gates mechanical allodynia. J Clin Invest.

[CR103] Luz LL, Szucs P, Safronov BV (2014). Peripherally driven low-threshold inhibitory inputs to lamina I local-circuit and projection neurones: a new circuit for gating pain responses. J Physiol.

[CR104] Luz LL, Fernandes EC, Dora F, Lukoyanov NV, Szucs P, Safronov BV (2019). Trigeminal adelta- and C-afferent supply of lamina I neurons in the trigeminocervical complex. Pain.

[CR105] Lynch ME, Watson CP (2006). The pharmacotherapy of chronic pain: a review. Pain Res Manag.

[CR106] Mar L, Yang FC, Ma Q (2012). Genetic marking and characterization of Tac2-expressing neurons in the central and peripheral nervous system. Mol Brain.

[CR107] Marshall GE, Shehab SA, Spike RC, Todd AJ (1996). Neurokinin-1 receptors on lumbar spinothalamic neurons in the rat. Neuroscience.

[CR109] Marvizon JC, Chen W, Murphy N (2009). Enkephalins, dynorphins, and beta-endorphin in the rat dorsal horn: an immunofluorescence colocalization study. J Comp Neurol.

[CR110] Matsushita M, Hosoya Y (1979). Cells of origin of the spinocerebellar tract in the rat, studied with the method of retrograde transport of horseradish peroxidase. Brain Res.

[CR111] Maxwell DJ, Belle MD, Cheunsuang O, Stewart A, Morris R (2007). Morphology of inhibitory and excitatory interneurons in superficial laminae of the rat dorsal horn. J Physiol.

[CR112] Melzack R, Wall PD (1965). Pain mechanisms: a new theory. Science.

[CR113] Menetrey D, Chaouch A, Binder D, Besson JM (1982). The origin of the spinomesencephalic tract in the rat: an anatomical study using the retrograde transport of horseradish peroxidase. J Comp Neurol.

[CR114] Merighi A (2018). The histology, physiology, neurochemistry and circuitry of the substantia gelatinosa Rolandi (lamina II) in mammalian spinal cord. Prog Neurobiol.

[CR115] Mermet-Joret N, Chatila N, Pereira B, Monconduit L, Dallel R, Antri M (2017). Lamina specific postnatal development of PKCγ interneurons within the rat medullary dorsal horn. Dev Neurobiol.

[CR116] Miraucourt LS, Dallel R, Voisin DL (2007). Glycine inhibitory dysfunction turns touch into pain through PKCγ interneurons. PLoS ONE.

[CR117] Miraucourt LS, Moisset X, Dallel R, Voisin DL (2009). Glycine inhibitory dysfunction induces a selectively dynamic, morphine-resistant, and neurokinin 1 receptor-independent mechanical allodynia. J Neurosci.

[CR118] Morisset V, Nagy F (1998). Nociceptive integration in the rat spinal cord: role of non-linear membrane properties of deep dorsal horn neurons. Eur J Neurosci.

[CR119] Naim M, Spike RC, Watt C, Shehab SA, Todd AJ (1997). Cells in laminae III and IV of the rat spinal cord that possess the neurokinin-1 receptor and have dorsally directed dendrites receive a major synaptic input from tachykinin-containing primary afferents. J Neurosci.

[CR120] Nelson TS, Fu W, Donahue RR, Corder GF, Hokfelt T, Wiley RG, Taylor BK (2019). Facilitation of neuropathic pain by the NPY Y1 receptor-expressing subpopulation of excitatory interneurons in the dorsal horn. Sci Rep.

[CR121] Nichols ML (1999). Transmission of chronic nociception by spinal neurons expressing the substance P receptor. Science.

[CR122] Oliveira AL (2003). Cellular localization of three vesicular glutamate transporter mRNAs and proteins in rat spinal cord and dorsal root ganglia. Synapse.

[CR123] Pagani M, Albisetti GW, Sivakumar N, Wildner H, Santello M, Johannssen HC, Zeilhofer HU (2019). How gastrin-releasing peptide opens the spinal gate for itch. Neuron.

[CR124] Paixão S, Loschek L, Gaitanos L, Alcalà Morales P, Goulding M, Klein R (2019). Identification of spinal neurons contributing to the dorsal column projection mediating fine touch and corrective motor movements. Neuron.

[CR125] Peirs C, Seal RP (2016). Neural circuits for pain: recent advances and current views. Science.

[CR126] Peirs C, Patil S, Bouali-Benazzouz R, Artola A, Landry M, Dallel R (2014). Protein kinase C gamma interneurons in the rat medullary dorsal horn: distribution and synaptic inputs to these neurons, and subcellular localization of the enzyme. J Comp Neurol.

[CR127] Peirs C (2015). Dorsal horn circuits for persistent mechanical pain. Neuron.

[CR128] Peirs C, Bourgois N, Artola A, Dallel R (2016). Protein kinase C gamma interneurons mediate c-fiber-induced orofacial secondary static mechanical allodynia, but not C-fiber-induced nociceptive behavior. Anesthesiology.

[CR129] Petitjean H (2015). Dorsal horn parvalbumin neurons are gate-keepers of touch-evoked pain after nerve injury. Cell Rep.

[CR130] Petitjean H (2019). Recruitment of spinoparabrachial neurons by dorsal horn calretinin neurons. Cell Rep.

[CR131] Polgar E, Fowler JH, McGill MM, Todd AJ (1999). The types of neuron which contain protein kinase C gamma in rat spinal cord. Brain Res.

[CR132] Polgar E, Shehab SA, Watt C, Todd AJ (1999). GABAergic neurons that contain neuropeptide Y selectively target cells with the neurokinin 1 receptor in laminae III and IV of the rat spinal cord. J Neurosci.

[CR133] Polgar E, Hughes DI, Riddell JS, Maxwell DJ, Puskar Z, Todd AJ (2003). Selective loss of spinal GABAergic or glycinergic neurons is not necessary for development of thermal hyperalgesia in the chronic constriction injury model of neuropathic pain. Pain.

[CR134] Polgar E, Furuta T, Kaneko T, Todd A (2006). Characterization of neurons that express preprotachykinin B in the dorsal horn of the rat spinal cord. Neuroscience.

[CR135] Polgar E, Thomson S, Maxwell DJ, Al-Khater K, Todd AJ (2007). A population of large neurons in laminae III and IV of the rat spinal cord that have long dorsal dendrites and lack the neurokinin 1 receptor. Eur J Neurosci.

[CR136] Polgar E, Al-Khater KM, Shehab S, Watanabe M, Todd AJ (2008). Large projection neurons in lamina I of the rat spinal cord that lack the neurokinin 1 receptor are densely innervated by VGLUT2-containing axons and possess GluR4-containing AMPA receptors. J Neurosci.

[CR137] Polgar E, Sardella TC, Watanabe M, Todd AJ (2011). Quantitative study of NPY-expressing GABAergic neurons and axons in rat spinal dorsal horn. J Comp Neurol.

[CR138] Polgar E, Durrieux C, Hughes DI, Todd AJ (2013). A quantitative study of inhibitory interneurons in laminae I–III of the mouse spinal dorsal horn. PLoS ONE.

[CR139] Polgar E, Sardella TC, Tiong SY, Locke S, Watanabe M, Todd AJ (2013). Functional differences between neurochemically defined populations of inhibitory interneurons in the rat spinal dorsal horn. Pain.

[CR140] Prescott SA, De Koninck Y (2002). Four cell types with distinctive membrane properties and morphologies in lamina I of the spinal dorsal horn of the adult rat. J Physiol.

[CR141] Procaccini C, De Rosa V, Pucino V, Formisano L, Matarese G (2015). Animal models of multiple sclerosis. Eur J Pharmacol.

[CR143] Punnakkal P, von Schoultz C, Haenraets K, Wildner H, Zeilhofer HU (2014). Morphological, biophysical and synaptic properties of glutamatergic neurons of the mouse spinal dorsal horn. J Physiol.

[CR144] Puskar Z, Polgar E, Todd AJ (2001). A population of large lamina I projection neurons with selective inhibitory input in rat spinal cord. Neuroscience.

[CR146] Ramón y Cajal S (1909). Histologie du système nerveux de l'homme and des vertébrés.

[CR147] Rexed B (1952). The cytoarchitectonic organization of the spinal cord in the cat. J Comp Neurol.

[CR148] Ribeiro-da-Silva A, De Koninck Y (2008) 5.23 - Morphological and neurochemical organization of the spinal dorsal horn. In: Masland RH et al. (eds) The senses: A comprehensive reference. Academic press, New York, pp 279–310. 10.1016/B978-012370880-9.00163-8

[CR150] Rowan S, Todd AJ, Spike RC (1993). Evidence that neuropeptide Y is present in GABAergic neurons in the superficial dorsal horn of the rat spinal cord. Neuroscience.

[CR151] Ruscheweyh R, Sandkuhler J (2002). Lamina-specific membrane and discharge properties of rat spinal dorsal horn neurones in vitro. J Physiol.

[CR152] Ruscheweyh R, Ikeda H, Heinke B, Sandkuhler J (2004). Distinctive membrane and discharge properties of rat spinal lamina I projection neurones in vitro. J Physiol.

[CR153] Sardella TC, Polgar E, Garzillo F, Furuta T, Kaneko T, Watanabe M, Todd AJ (2011). Dynorphin is expressed primarily by GABAergic neurons that contain galanin in the rat dorsal horn. Mol Pain.

[CR154] Sardella TC, Polgar E, Watanabe M, Todd AJ (2011). A quantitative study of neuronal nitric oxide synthase expression in laminae I-III of the rat spinal dorsal horn. Neuroscience.

[CR155] Sathyamurthy A (2018). Massively parallel single nucleus transcriptional profiling defines spinal cord neurons and their activity during behavior. Cell Rep.

[CR156] Scheibel ME, Scheibel AB (1968). Terminal axonal patterns in cat spinal cord II. The dorsal horn. Brain Res.

[CR157] Schoenen J (1982). The dendritic organization of the human spinal cord: the dorsal horn. Neuroscience.

[CR158] Sengul G, Watson C, Paxinos G (2015). Chapter 8 - ascending and descending pathways in the spinal cord. The rat nervous system.

[CR159] Simmons DR, Spike RC, Todd AJ (1995). Galanin is contained in GABAergic neurons in the rat spinal dorsal horn. Neurosci Lett.

[CR160] Smith KM (2015). Functional heterogeneity of calretinin-expressing neurons in the mouse superficial dorsal horn: implications for spinal pain processing. J Physiol.

[CR161] Smith KM, Boyle KA, Mustapa M, Jobling P, Callister RJ, Hughes DI, Graham BA (2016). Distinct forms of synaptic inhibition and neuromodulation regulate calretinin-positive neuron excitability in the spinal cord dorsal horn. Neuroscience.

[CR164] Spike RC, Puskar Z, Andrew D, Todd AJ (2003). A quantitative and morphological study of projection neurons in lamina I of the rat lumbar spinal cord. Eur J Neurosci.

[CR165] Sun YG, Chen ZF (2007). A gastrin-releasing peptide receptor mediates the itch sensation in the spinal cord. Nature.

[CR166] Sun YG, Zhao ZQ, Meng XL, Yin J, Liu XY, Chen ZF (2009). Cellular basis of itch sensation. Science.

[CR167] Sun S, Xu Q, Guo C, Guan Y, Liu Q, Dong X (2017). Leaky gate model: intensity-dependent coding of pain and itch in the spinal cord. Neuron.

[CR168] Tansley SN, Wong C, Uttam S, Mogil JS, Khoutorsky A (2018). Translation regulation in the spinal dorsal horn—a key mechanism for development of chronic pain. Neurobiol Pain.

[CR169] Tiong SY, Polgar E, van Kralingen JC, Watanabe M, Todd AJ (2011). Galanin-immunoreactivity identifies a distinct population of inhibitory interneurons in laminae I–III of the rat spinal cord. Mol Pain.

[CR170] Todd AJ (2017). Identifying functional populations among the interneurons in laminae I–III of the spinal dorsal horn. Mol Pain.

[CR171] Todd AJ, Lewis SG (1986). The morphology of Golgi-stained neurons in lamina II of the rat spinal cord. J Anat.

[CR172] Todd AJ, McKenzie J (1989). GABA-immunoreactive neurons in the dorsal horn of the rat spinal cord. Neuroscience.

[CR173] Todd AJ, Sullivan AC (1990). Light microscope study of the coexistence of GABA-like and glycine-like immunoreactivities in the spinal cord of the rat. J Comp Neurol.

[CR175] Todd AJ, Spike RC, Polgar E (1998). A quantitative study of neurons which express neurokinin-1 or somatostatin sst2a receptor in rat spinal dorsal horn. Neuroscience.

[CR176] Todd AJ, McGill MM, Shehab SA (2000). Neurokinin 1 receptor expression by neurons in laminae I, III and IV of the rat spinal dorsal horn that project to the brainstem. Eur J Neurosci.

[CR177] Todd AJ, Hughes DI, Polgar E, Nagy GG, Mackie M, Ottersen OP, Maxwell DJ (2003). The expression of vesicular glutamate transporters VGLUT1 and VGLUT2 in neurochemically defined axonal populations in the rat spinal cord with emphasis on the dorsal horn. Eur J Neurosci.

[CR178] Truex RC, Taylor MJ, Smythe MQ, Gildenberg PL (1970). The lateral cervical nucleus of cat, dog and man. J Comp Neurol.

[CR179] Uttam S (2018). Translational profiling of dorsal root ganglia and spinal cord in a mouse model of neuropathic pain. Neurobiol Pain.

[CR180] Vardeh D, Mannion RJ, Woolf CJ (2016). Toward a mechanism-based approach to pain diagnosis. J Pain.

[CR181] Wang X (2013). Excitatory superficial dorsal horn interneurons are functionally heterogeneous and required for the full behavioral expression of pain and itch. Neuron.

[CR182] Wang D, Tawfik VL, Corder G, Low SA, Francois A, Basbaum AI, Scherrer G (2018). Functional divergence of delta and Mu opioid receptor organization in CNS pain circuits. Neuron.

[CR183] Wickersham IR (2007). Monosynaptic restriction of transsynaptic tracing from single, genetically targeted neurons. Neuron.

[CR184] Willis WD, Coggeshall RE, Willis WD, Coggeshall RE (1978). Structure of the dorsal horn. Sensory mechanisms of the spinal cord.

[CR186] Willis WD, Kenshalo DR, Leonard RB (1979). The cells of origin of the primate spinothalamic tract. J Comp Neurol.

[CR187] Xu Q, Yaksh TL (2011). A brief comparison of the pathophysiology of inflammatory versus neuropathic pain. Curr Opin Anaesthesiol.

[CR189] Yasaka T, Tiong SY, Hughes DI, Riddell JS, Todd AJ (2010). Populations of inhibitory and excitatory interneurons in lamina II of the adult rat spinal dorsal horn revealed by a combined electrophysiological and anatomical approach. Pain.

[CR191] Yuengert R (2015). Origin of a Non-clarke’s column division of the dorsal spinocerebellar tract and the role of caudal proprioceptive neurons in motor function. Cell Rep.

[CR192] Zeilhofer HU, Benke D, Yevenes GE (2012). Chronic pain states: pharmacological strategies to restore diminished inhibitory spinal pain control. Annu Rev Pharmacol Toxicol.

[CR193] Zeilhofer HU, Wildner H, Yevenes GE (2012). Fast synaptic inhibition in spinal sensory processing and pain control. Physiol Rev.

[CR194] Zeisel A (2018). Molecular architecture of the mouse nervous system. Cell.

[CR195] Zhang ET, Han ZS, Craig AD (1996). Morphological classes of spinothalamic lamina I neurons in the cat. J Comp Neurol.

[CR196] Zhang MD (2014). Neuronal calcium-binding proteins 1/2 localize to dorsal root ganglia and excitatory spinal neurons and are regulated by nerve injury. Proc Natl Acad Sci USA.

[CR197] Zhang MD (2016). Comparative anatomical distribution of neuronal calcium-binding protein (NECAB) 1 and -2 in rodent and human spinal cord. Brain Struct Funct.

[CR198] Zhang Y, Liu S, Zhang YQ, Goulding M, Wang YQ, Ma Q (2018). Timing mechanisms underlying gate control by feedforward inhibition. Neuron.

[CR199] Zou W, Song Z, Guo Q, Liu C, Zhang Z, Zhang Y (2011). Intrathecal lentiviral-mediated RNA interference targeting PKCgamma attenuates chronic constriction injury-induced neuropathic pain in rats. Hum Gene Ther.

