# Correction to: Recent advances in our understanding of the organization of dorsal horn neuron populations and their contribution to cutaneous mechanical allodynia

**DOI:** 10.1007/s00702-020-02250-7

**Published:** 2020-09-22

**Authors:** Cedric Peirs, Radhouane Dallel, Andrew J. Todd

**Affiliations:** 1 Université Clermont Auvergne, CHU Clermont-Ferrand, Inserm, Neuro-Dol, Clermont-Ferrand, F-63000 France; 2 Institute of Neuroscience and Psychology, College of Medical, Veterinary and Life Sciences, University of Glasgow, Glasgow, G12 8QQ UK

## Correction to: Journal of Neural Transmission (2020) 127:505–525 10.1007/s00702-020-02159-1

The original version of this article unfortunately contained a mistake. The affiliation “2” (Institute of Neuroscience and Psychology, College of Medical, Veterinary and Life Sciences, University of Glasgow, Glasgow G12 8QQ, UK) has been assigned to all authors. It should be assigned to Andrew J. Todd only.

List of authors should read as: Cedric Peirs^1^ · Radhouane Dallel^1^ · Andrew J. Todd^2^.

