# Supplementary material for: Recent advances in our understanding of the organization of dorsal horn neuron populations and their contribution to cutaneous mechanical allodynia
Source: J Neural Transm (Vienna). 2020 Apr 2;127(4):505–25. doi: 10.1007/s00702-020-02159-1 (PMC7148279; doi:10.1007/s00702-020-02159-1)
Supplement: Supplementary file 1 — Supplementary file1 (PDF 1834 kb) [file 702_2020_2159_MOESM1_ESM.pdf]

Recent advances in our understanding of the organization of dorsal horn neuron populations and their contribution to cutaneous mechanical allodynia

Cedric Peirs [1*], Radhouane Dallel [1] and Andrew J. Todd [2]

[1] Université Clermont Auvergne, CHU Clermont-Ferrand, Inserm, Neuro-Dol.
[2] Institute of Neuroscience and Psychology, College of Medical, Veterinary and Life Sciences, University of Glasgow, Glasgow G12 8QQ, UK

Electronic Supplementary Material:

Number of Figures: 4 tables

*Corresponding author: Cedric Peirs, Neuro-Dol, Inserm U1107, Faculty of Dental Surgery, 63100 Clermont-Ferrand, France. Email: cedric.peirs@inserm.fr

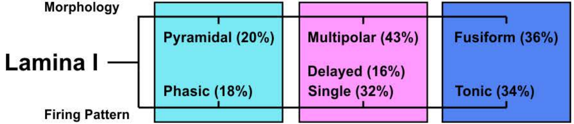

Projection Neurons

% of lamina	5%-11%
Expression of other markers	NK1R 80%-90%<br>MOR 86%<br>DOR 56%<br>SP 16%<br>SST2A 9/10-15%<br>DYN 1%<br>GRP 0%<br>GRPR 0%

Excitatory / Inhibitory

Marker	NK1R	NKB	MOR1	PKCγ	SOM	CCK	DYN	GABA	SST2A	GLY	GAL	NPY	NNOS
Expression	Adult	Adult	Adult	Adult	Adult	Adult	Adult	Adult	Adult	Adult	Adult	Adult	Adult
% of lamina	19-45%	11%	11%	8%	0-7%	7%	17%	25/31-36%	13-17%	12%	7%	6%	4%
Expression of other markers	PKCγ 22%<br>NKB 3%<br>SST2A 0-2%<br>SOM 0%<br>GABA 0%<br>GRP 0%<br>GRPR 0%	GAD 0%		GABA 0%<br>GLY 0%<br>NNOS 0%	SST2A 0%<br>NK1R 0%		GABA 47%<br>GAL 47%<br>NNOS 0%<br>PKCγ 0%	GLY 33-47%<br>NK1R 0%	GABA 100%-100%<br>GLY 83%<br>NK1R 0-1%<br>SOM 0%	GABA100%<br>PKCγ 0%	GABA 100%<br>SST2A 100%<br>DYN 96%<br>NPY 0%<br>NNOS 0%	GABA 100%-100%<br>GLY 6%<br>GAL 0%	GABA 100%<br>PKCγ 0%<br>GAL 0%<br>DYN 0%

ESM_1 Populations of dorsal horn neurons in lamina I for which quantitative expression within laminae is available. Lamina I neurons can be sorted based on their morphology and firing patterns (upper schematic). Neurons that are pyramidal have phasing firing (blue square), those that are multipolar have delayed or single firing (pink square), and the last class of fusiform neurons are mostly tonic (dark blue square). Percentages represent the proportion of neurons within the lamina that belong to each population. Lamina I neurons can also be organized based on their molecular profile. Using such criteria, populations of neurons that are mostly excitatory are displayed in light blue and those that are mostly inhibitory neurons are displayed in light pink. A population that is equally composed of excitatory and inhibitory neurons is represented in grey. Projection neurons of lamina I that send axons to supraspinal areas are represented in green. Range of values correspond to the minimum and maximum percentage of neurons that express a marker reported for each population. Values obtained in mice are shown in black and those obtained in rats are displayed in brown. References used in this table can be find in ESM_4

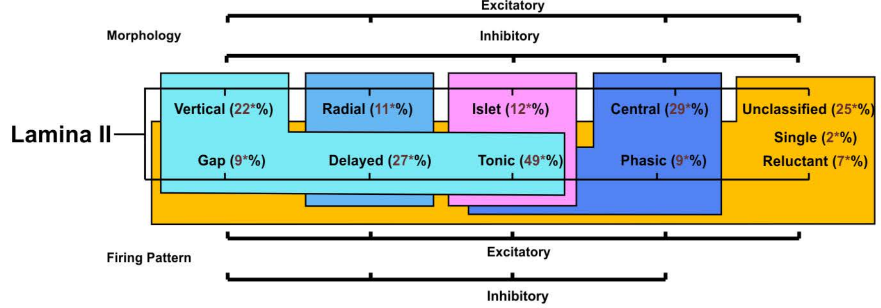

Excitatory

Marker	SOM	CR	PKCγ	MOR1	SP	GRP	NKB	CCK	NTS
Expression	Adult	Adult	Adult	Adult	Adult	Adult	Adult	Adult	Adult
% of lamina	37*%-45%	39%	19*%-21%	20*%	14-20%	11-14%	10*%-12%	5-7*%	7%
Morphology	V, R, C	V, R, I, C	R 55%<br>C 26%<br>V 7%<br>I 5%		R	C 84%<br>V 3%			
Firing pattern	D 45%<br>S 17%<br>P 32%	D 62%<br>S 24%<br>T 5%<br>P 5%<br>R 5%	T 46%<br>P 16%<br>G 16%<br>S 21%		D 79%<br>T 8%<br>S 5%<br>P 3%<br>G 3%<br>R 2%	P 49-94%<br>S 0-33%<br>T 6-8%<br>R 0-6%<br>D 0-3%			
Expression of other markers	VGLUT2 94%<br>CR 53%<br>CB 46%<br>PKCγ 35%/45%<br>tVGLUT3 28%<br>NKB 21%<br>GRP 15%<br>NTS 10%<br>GAD67 2%<br>GABA 0%	SOM 67%<br>PKCγ 22%<br>NKB 21%<br>GRP 19%<br>PAX2 15-17%<br>VGLUT3 7-45%<br>NTS 0%<br>NECAB1 45%	NECAB2 95-89%<br>SOM 74%<br>NECAB1 0-55%<br>CR 50%<br>CCK 47%<br>NTS 31%<br>NKB 29-31%<br>VGLUT3 25%<br>GAD67 20-24*%<br>GABA 8*%<br>GRP 5-11%<br>SP 4%<br>NPYY1R 0%<br>PV 0%<br>NNOS 0%	PKCγ 5%<br>GABA 5%	CB 44%<br>SOM 57%<br>PAX2 12-19%<br>GRP 0-11%<br>CCK 3%<br>PKCγ 2%	VGLUT2 100%<br>LMX1B 74-86%<br>TLX3 70-79%<br>SOM 59%<br>CR 55%<br>CB 21-29%<br>PKCγ 3-17%<br>SP 0-12%<br>NPFF 0-6%<br>NTS 1%<br>NKB 0%<br>CCK 0%<br>VGAT 0%<br>PAX2 0%	SOM 31-91%<br>TLX3 85%<br>CR 84%<br>CB 84%<br>PKCγ 20*-76%<br>NTS 0-6%<br>NK1R 5%<br>CCK 2%<br>PAX2 2%<br>MOR1 1%<br>GRP 0%<br>NPFF 0%<br>GAD 0*%	VGLUT2 93%<br>PKCγ 47%<br>SP 21-29%<br>NTS 1%<br>NKB 1%<br>GRP 0%<br>PAX2 0%	PKCγ 76-98%<br>CB 75%<br>SOM 73%<br>NKB 0-13%<br>TRH 4%<br>GRP 2%<br>CR 0%<br>GABA 0*%

Marker	NPFF	NK1R	DOR	GRPR	NPYY1R	CB	TRH	NECAB1	NECAB2
Expression	Adult	Adult	Adult	Adult	Adult	Adult	Adult	Adult	Adult
% of lamina	5%	5*-6*%							
Morphology									
Firing pattern			D D 35%<br>S 25%<br>G 20%<br>T 15%<br>P 5%	D 56-58%<br>T 21-23%<br>P 15%<br>G 0-7%<br>S 0-5%					
Expression of other markers	SOM 85%<br>SP 5%<br>CCK 1%<br>NTS 1%<br>GRP 0-33%<br>NKB 0%<br>PAX2 0%	GABA 0%<br>SST2A 0-0%<br>GRPR 0%	VGLUT2 85%<br>SOM 62%<br>CB 50%<br>CR 27%<br>VGAT 25%<br>NNOS 12%<br>PAX2 8%<br>PKCγ 7%	VGLUT2 63-81%<br>LMX1B 62%<br>PAX2 27%<br>VGAT 19%<br>NK1R 0%	CALB 36%<br>CR 18%<br>PAX2 2%<br>PKCγ 0%<br>PV 0*%	NTS 13%<br>SOM 11%<br>SP 10%<br>NECAB1 48%	PKCγ 91%<br>CCK 85%<br>NTS 18%	VGLUT2 72%<br>CB 38%<br>CR 33%<br>PKCγ 0-12%<br>SST2A 2-11%<br>PV 5%	VGLUT2 90%<br>GAD67 0-0%

Inhibitory

Marker	GABA	PAX2	NNOS	SST2A	DYN	GLY	NPY	PRP	GAL	PV	BHLHB5
Expression	Adult	Adult	Adult	Adult	Adult	Adult	Adult	Adult	Adult	Adult	Development
% of lamina	24*%-46*%	25%	18*%	14-14%	4*-14%	12*%	5*-8%	3/4*-7%	3%		
Morphology	I 43%<br>C 14%<br>V 11%				V		I 0%	C 100%		C-I 82%	C
Firing pattern	T 83%<br>P 13%<br>G 4%<br>D 4%				T, P		T 84%<br>P 8%<br>S 7%	T 72-93<br>P 13%<br>S 7%		T 71%<br>P 29%	
Expression of other markers	PAX2 100*%<br>GLY 37*-43*%<br>SOM 0%<br>NTS 0*%<br>NK1R 0%	GABA 100%<br>CR 27%<br>GRP 0%<br>CCK 0%<br>NPFF 0%	SST2A 57%<br>GABA 33*%<br>PKCγ 0-32*%<br>PV 0%<br>GAL 0%<br>DYN 0%	GABA 99-100%<br>GLY 83%<br>NECAB1 15-54%<br>GAL 26-44%<br>NNOS 30-36%<br>PRP 29%<br>NECAB2 7%<br>NK1R 0-1%<br>PV 0%	GAD67 86%<br>GABA 69-79*%<br>PAX2 67-73%<br>SST2A 72%<br>GAL 70*%<br>GLYT2 28%<br>VGLUT2 12%<br>NPY 7-9%<br>PV 0*%<br>NNOS 0%	GABA 99*-100%	GABA 100*%<br>PAX2 100%<br>SST2A 15-24%<br>DYN 13%<br>GLY 6*%<br>NNOS 4%<br>BHLHB5 2%<br>GAL 0%	SST2A 100%<br>GABA 80%<br>NNOS 70%<br>GAL 28%<br>NPY 0%<br>PV 0%	GABA 100%<br>SST2A 97-100%<br>BHLHB5 95%<br>DYN 92*%<br>PRP 41%<br>PV 0%<br>NNOS 0%<br>NPY 0%	GABA 70-75%<br>NECAB1 31%<br>NPY 4%<br>SST2A 0-1%<br>PKCγ 0%<br>GAL 0%<br>DYN 0*%<br>NNOS 0%<br>NPYYR1 0*%	LMX1B 30%<br>PAX2 70%<br>GAL 78%

ESM_2 Populations of dorsal horn neurons in lamina II for which quantitative expression within laminae is available. Lamina II neurons can be sorted based on their morphology and firing patterns (upper schematic). Vertical cells have gap, delayed or tonic firing (blue square), radial cells have delayed firing (medium blue square), islet cells are invariable tonic (pink square) and central cells have either tonic or phasic firing (dark blue square). A last class of lamina II neurons for which morphology does not fit usual categories can display all firing pattern that exist in this lamina. Percentages represent the proportion of neurons within the lamina that belong to each population. Lamina II neurons can also be organized based on their molecular profile. Using such criteria, populations of neurons that are mostly excitatory are displayed in light blue and those that are mostly inhibitory neurons are displayed in light pink. Range of values correspond to the minimum and maximum percentage of neurons that express a marker reported for each population. Values obtained in mice are shown in black and those obtained in rats are displayed in brown. Data that were obtained exclusively in lamina II are shown with an asterisk. Other data include analysis of both lamina I and lamina II. References used in this table can be find in ESM_4

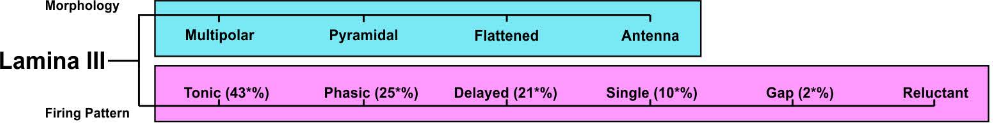

Excitatory

Marker	VGLUT3	SOM	CCK	PKCγ	NK1R	CBLN2	IGFBP5	HTR6	NTS	NKB	SP	DYN
Expression	Development	Adult	Adult	Adult	Adult	Adult	Adult	Adult	Adult	Adult	Adult	Adult
% of lamina	32%	16-17%	3*-15%	6-14%	6-11%	11*%	11*%	10*%	8%	4-4%	4%	1-4%
Firing pattern	P 17-58%<br>T 8-54%<br>D 0-66%<br>S 7-9%		P 67*%<br>T 33*%	P 29*%<br>D 56*%<br>T 14*%		P48*%<br>S 25*%<br>T 21*%	T 76*%<br>R 22*%	R 76*%<br>T 10*%<br>S 10*%				
Expression of other markers	VGLUT2 96%<br>PKCγ 16-42%<br>SOM 38%<br>CR 10%<br>NK1R 1%	PKCγ 50%<br>NKB 25%<br>GLYT2 5%<br>NTS 3%	VGLUT2 92-98*%<br>SP 13-21%<br>NEUD4 4*%<br>PKCγ 1*%<br>GRP 1%<br>NKB 1%<br>NTS 1%<br>CBLN2 1*%<br>HTR6 1*%<br>VGAT 1*%<br>IGFPB5 0*%<br>PV 0*%<br>PAX2 0%	VGLUT2 97*%<br>NEUD4 91*%<br>SOM 56%<br>CCK 4*-47%<br>NTS 45<br>NKB 13%<br>GABA 8%<br>IGFPB5 3*%<br>CBLN2 2*%<br>HTR6 2*%<br>VGAT 1*%<br>SP 0%<br>GLY 0%<br>PV 0*%	PKCγ 37%<br>GABA 10%	VGLUT2 81*%<br>VGAT 5*%<br>CCK* 2%<br>HTR6* 8%<br>PKCγ* 3%<br>PV 0*%	VGLUT2 73*%<br>VGAT 15*%<br>CCK 2*%<br>HTR6 4*%<br>PKCγ 3*%	VGLUT2 88*%<br>VGAT 6*%<br>CCK 4*%<br>PKCγ 3*%<br>NEUD4 2*%<br>CBLN2 1*%<br>IGFBP5 1*%	PKCγ 75-79%<br>NKB 0-34%<br>SOM 10%<br>TRH 4%<br>GABA 0%	SOM 55%<br>PKCγ 46%<br>NTS 0-30%<br>CCK 2%<br>GRP 0%<br>GAD67 0%	CCK 10-40%<br>PAX2 21%<br>GRP 0%<br>PKCγ 0%	GABA 0%<br>PV 0%<br>GAL 0%

Marker	NEUD4	LMX1B	RORα	MAFA	MAFB	CMAF	LBX1	TRH	NECAB1	CB	CR	GRP
Expression	Adult	Adult	Adult	Adult	Adult	Adult	Adult	Adult	Adult	Adult	Adult	Adult
% of lamina	2*%											
Firing pattern	T 100*%											
Expression of other markers	VGLUT2 85*%<br>PKCγ 18*%<br>HTR6 5*%<br>CCK 4*%<br>VGAT 3*%	PAX2 0%<br>GBX1 0%<br>GAD67 0%	VGLUT2 92%<br>LMX1B 86-92%<br>CCK 60%<br>CMAF 49%<br>MAFA 48%<br>PKCγ 22%<br>GAD67 3%<br>GBX1 3%<br>PAX2 1%	LMX1B 93%<br>GAD67 0%<br>GBX1 0%	LMX1B 62%<br>PAX2 29%<br>GBX1 19%<br>GAD67 13%	LMX1B 62%<br>PAX2 29%<br>GBX1 19%<br>GAD67 18%	LMX1B 79%<br>GBX1 21%<br>PAX2 18%<br>GAD67 11%	PKCγ 91%<br>CCK 85%<br>NTS 18%	CB 25%<br>PV 16%<br>CR 8%	NECAB1 23%	NECAB1 18%	NKB 0%<br>SP 0%

Inhibitory

Marker	PAX2	GABA	GLY	PV	NPY	KCNIP2	SST2A	CDH3	GAL	RET	GBX1	GAD67
Expression	Adult	Adult	Adult	Adult	Adult	Adult	Adult	Adult	Adult	Development	Adult	Adult
% of lamina	40%	30-53%	20-36%	13*%	4-9%	9*%	6%	5*%	2%			
Firing pattern				T 100*%	T 84%<br>P 8%<br>S 7%	D 100*%		G 46*%<br>T 38*%<br>P 8*%<br>D 7*%		T 64-80%<br>D 8-26%<br>P 3-4%<br>S 3-8%		
Expression of other markers	GABA 100%<br>GAD67 82%<br>GBX1 81%<br>LMX1B 0%<br>CCK 0%	PAX2 100%<br>GLY 64-89%<br>NTS 0%<br>DYN 0%	GABA 96-98%<br>PKCγ 0%	GABA 70-75%<br>VGAT 63*%<br>VGLUT2 35*%<br>CDH3 35*%<br>NECAB1 17%<br>IGFBP5 2*%<br>NPY 2%<br>RORβ 1*%<br>NEUD4 1*%<br>HTR6 1*%<br>CBLN2 0*%<br>KCNIP2 0*%<br>CCK 0*%<br>PKCγ 0*%<br>GAL 0%<br>SST2A 0%<br>NNOS 0-0%<br>DYN 0%	GABA 100%<br>PAX2 100%<br>NNOS 1-6%<br>SST2A 4%<br>GAL 0%	VGAT 92*%<br>VGLUT2 7*%<br>CDH3 1*%<br>RORβ 1*%<br>PV 0*%	GABA 50-92%<br>PV 0%	VGAT 79*%<br>VGLUT2 8*%<br>RORβ 3*%<br>KCNIP2 2*%	GABA 100%<br>NNOS 83%<br>SST2A 10%<br>GLY 1%<br>DYN 0%<br>PV 0%<br>NPY 0%	VGAT 91-93%<br>GLYT2 51%<br>GAD67 47%<br>PV 24-31%<br>VGLUT2 11%	PAX2 87-95%<br>GAD67 77%<br>LMX1B 0%<br>MAFA 0%	PAX2 82%<br>GBX1 77%<br>RORβ 32%<br>CMAF 18%<br>MAFB 13%<br>LBX1 11%<br>LMX1B 0%<br>MAFA 0%<br>NKB 0%

Mixte

Marker	RORβ	NNOS
Expression	Adult	Adult
% of lamina	8*%	5%
Firing pattern	D 70*%<br>T 29*%	
Expression of other markers	VGAT 80*%<br>PAX2 54-58%<br>GBX1 51%<br>LMX1B 48%<br>VGLUT2 11*-43%<br>GAD67 32%<br>NNOS 21%<br>GAD65 20%<br>DYN 11%<br>GAL 9%<br>PV 6%<br>NPY 5%<br>CDH3 1*%<br>KCNIP2 0*%	PKCγ 53%<br>GABA 50%<br>SST2A 29%<br>PV 0%<br>NPY 0%

Lamina IV-VI

Marker	ZIC2	ATOH1	SATB1/2	TCFAP2B
Expression	Development	Development	Development	Development
% of lamina				
Firing pattern				
Expression of other markers	LMX1B 64%<br>RORα 28%<br>SATB1/2 16%<br>TCFAP2B 15%<br>PAX2 4%	VGLUT2 82%	TLX3 52%<br>PAX2 22%	GAD65 67%<br>GAD67 40%<br>VGLUT2 26%

Projection Neurons

% of lamina	2%
Expression of other markers	NK1R 33-44%

ESM_3 Populations of dorsal horn neurons in lamina III for which quantitative expression within laminae is available. Lamina III neurons can be sorted based on their morphology and firing patterns but no correlation has been established between these two criteria (upper schematic). Lamina III cells can have multipolar, pyramidal, flattened or antenna-type morphologies. Their firing pattern can be tonic, phasic, delayed, single, gap or reluctant. Percentages represent the proportion of neurons within the lamina that belong to each population. Lamina II neurons can also be organized based on their molecular profile. Using such criteria, populations of neurons that are mostly excitatory are displayed in light blue and those that are mostly inhibitory neurons are displayed in light pink. Populations that are equally composed of excitatory and inhibitory neurons are represented in grey. Other populations of the deep dorsal horn are displayed in yellow. Projection neurons of lamina III that send axons to supraspinal areas are represented in green. Range of values correspond to the minimum and maximum percentage of neurons that express a marker reported for each population. Values obtained in mice are shown in black and those obtained in rats are displayed in brown. Data marked by an asterisk include neurons from lamina II_i to lamina III. Other data were obtained from analysis of lamina III exclusively. References used in this table can be find in ESM_4

Marker	References
ATOH1	(Yuengert et al. 2015)
BHLHB5	(Kardon et al. 2014; Ross et al. 2010)
CB	(Yoshida et al. 1990)
CBLN2	(Abraira et al. 2017)
CCK	(Abraira et al. 2017; Gutierrez-Mecinas et al. 2019b; Xu et al. 2008)
CDH3	(Abraira et al. 2017)
CMAF	(Del Barrio et al. 2013)
CR	(Cheng et al. 2017; Gutierrez-Mecinas et al. 2019c; Peirs et al. 2015; Smith et al. 2015; Smith et al. 2016; Zhang et al. 2014)
DYN	(Boyle et al. 2017; Duan et al. 2014; Huang et al. 2018; Sardella et al. 2011a; Xu et al. 2008)
DOR	(Wang et al. 2018)
GABA	(Abraira et al. 2017; Larsson 2017; Polgar et al. 2013a; Polgar et al. 2003; Polgar et al. 2013b; Todd and Sullivan 1990; Yasaka et al. 2010)
GAD67	(Del Barrio et al. 2013)
GAL	(Iwagaki et al. 2013; Kardon et al. 2014; Polgar et al. 2013b; Sardella et al. 2011a; Simmons et al. 1995; Tiong et al. 2011)
GBX1	(Del Barrio et al. 2013)
GLY	(Abraira et al. 2017; Polgar et al. 2013a; Polgar et al. 2003; Todd and Sullivan 1990)
GRP	(Albisetti et al. 2019; Dickie et al. 2018; Gutierrez-Mecinas et al. 2016; Gutierrez-Mecinas et al. 2014; Pagani et al. 2019; Solorzano et al. 2015)
GRPR	(Aresh et al. 2017; Bardoni et al. 2019)
HTR6	(Abraira et al. 2017)
KCNIP2	(Abraira et al. 2017)
LBX1	(Gross et al. 2002)
IGFBP5	(Abraira et al. 2017)
LMX1B	(Del Barrio et al. 2013; Gross et al. 2002; Qiu et al. 2009)
MAFA	(Del Barrio et al. 2013)
MAFB	(Del Barrio et al. 2013)
MOR1	(Kemp et al. 1996; Polgar et al. 1999; Spike et al. 2002)
NECAB1	(Zhang et al. 2016; Zhang et al. 2014)
NECAB2	(Zhang et al. 2016; Zhang et al. 2014)
NEUD4	(Abraira et al. 2017)
NK1R	(Bardoni et al. 2019; Duan et al. 2014; Littlewood et al. 1995; Polgar et al. 2013a; Polgar et al. 1999; Polgar et al. 2006; Todd et al. 1998)
NKB	(Gutierrez-Mecinas et al. 2016; Mar et al. 2012; Polgar et al. 2006)
NNOS	(Huang et al. 2018; Iwagaki et al. 2013; Martin et al. 1999; Polgar et al. 2013a; Polgar et al. 2013b; Sardella et al. 2011b; Tiong et al. 2011)
NPFF	(Gutierrez-Mecinas et al. 2019a)
NPY	(Boyle et al. 2017; Iwagaki et al. 2013; Kardon et al. 2014; Polgar et al. 2013b; Polgar et al. 2011; Rowan et al. 1993; Tiong et al. 2011)
NPYY1R	(Nelson et al. 2019)
NTS	(Gutierrez-Mecinas et al. 2019b; Gutierrez-Mecinas et al. 2016; Polgar et al. 1999; Proudlock et al. 1993; Todd et al. 1992)
PAX2	(Del Barrio et al. 2013; Gross et al. 2002; Gutierrez-Mecinas et al. 2017; Larsson 2017)
PKCγ	(Abraira et al. 2017; Alba-Delgado et al. 2015; Dickie et al. 2018; Gutierrez-Mecinas et al. 2019b; Gutierrez-Mecinas et al. 2019c; Gutierrez-Mecinas et al. 2016; Martin et al. 1999; Peirs et al. 2014; Peirs et al. 2015; Polgar et al. 1999; Sardella et al. 2011a; Sardella et al. 2011b; Solorzano et al. 2015; Zhang et al. 2016)
PRP	(Hantman et al. 2004; Iwagaki et al. 2013)
PV	(Abraira et al. 2017; Boyle et al. 2019; Boyle et al. 2017; Hughes et al. 2012; Laing et al. 1994; Martin et al. 1999; Polgar et al. 2013b; Tiong et al. 2011; Yoshida et al. 1990)
RET	(Cui et al. 2016)
RORα	(Bourane et al. 2015; Del Barrio et al. 2013; Paixão et al. 2019)
RORβ	(Abraira et al. 2017; Del Barrio et al. 2013; Koch et al. 2017)
SATB1/2	(Levine et al. 2014)
SP	(Dickie et al. 2018; Gutierrez-Mecinas et al. 2017; Gutierrez-Mecinas et al. 2019b; Gutierrez-Mecinas et al. 2019c; Xu et al. 2008)
SOM	(Cheng et al. 2017; Duan et al. 2014; Gutierrez-Mecinas et al. 2016; Proudlock et al. 1993)
SST2A	(Cameron et al. 2015; Iwagaki et al. 2013; Polgar et al. 2013a; Todd et al. 1998; Zhang et al. 2016)
TCFAP2B	(Levine et al. 2014)
TRH	(Gutierrez-Mecinas et al. 2019b)
VGLUT2	(Abraira et al. 2017; Gutierrez-Mecinas et al. 2019b; Gutierrez-Mecinas et al. 2019c; Wang et al. 2018)
VGLUT3	(Cheng et al. 2017; Peirs et al. 2015)
ZIC2	(Paixão et al. 2019)
Projection neurons	(Abraira et al. 2017; Bardoni et al. 2019; Bice and Beal 1997; Cameron et al. 2015; Gamboa-Esteves et al. 2004; Grudt and Perl 2002; Gutierrez-Mecinas et al. 2019a; Marshall et al. 1996; Polgar et al. 1999; Solorzano et al. 2015; Spike et al. 2003; Todd et al. 2000; Wang et al. 2018; Yuengert et al. 2015)

ESM_4 References used in ESM_1-3 for each dorsal horn population for which quantitative expression within laminae is available. Studies performed in mice are shown in black and those performed in rats are displayed in brown. ATOH1, Atonal Basic helix-loop-helix Transcription Factor 1; BHLHB5, Basic helix-loop-helix Domain Containing, Class B, 5; CB, Calbindin, CBLN2, Cerebellin-2; CCK, Cholecystokinin; CDH3, Cadherin-3; CMAF, Proto-Oncogene C-Maf, CR, Calretinin; DYN, Dynorphin; DOR, Opioid receptor δ; GABA, γ-aminobutyric acid; GAD67, Glutamic acid decarboxylase 67; GAL, Galanin; GBX1, Gastrulation Brain Homeobox 1; GLY, Glycine; GRP, Gastrin releasing peptide; GRPR, Gastrin-releasing peptide receptor; HTR6, Serotonin Receptor 6; KCNIP2, Kv Channel Interacting protein-2; LBX1, Ladybird homeobox 1; IGFBP5, Insulin-like Growth Factor Binding Protein 5; LMXB, LIM homeobox transcription factor 1 beta; MAFA, V-maf musculoaponeurotic fibrosarcoma oncogene homolog A; MAFB, V-maf musculoaponeurotic fibrosarcoma oncogene homolog B; MOR1, Opioid receptor μ 1; NECAB1, Neuronal calcium-binding protein 1; NECAB2, Neuronal calcium-binding protein 2; NEUD4, Neurogenic Differentiation Factor-4; NK1R, Neurokinin 1 receptor; NKB, Neurokinin B; NNOS, Neuronal nitric oxide synthase; NPFF, Neuropeptide FF; NPY, Neuropeptide Y; NPYY1R, Neuropeptide Y Y1 receptor; NTS, Neurotensin; PAX2, Paired box gene 2; PKCγ, Gamma isoform of protein kinase C; PRP, Prion promoter; PV, Parvalbumin; RET, Receptor tyrosine kinase; RORα, ROR α nuclear orphan receptor; RORβ, ROR β nuclear orphan receptor; SATB1/2, Special AT-rich sequence-binding proteins 1 and 2; SP, Substance P; SOM, Somatostatin; SST2A, Somatostatin receptor subtype 2A; TCFAP2B, Transcription factor AP-2 beta; TRH, Thyrotropin Releasing Hormone; VGLUT2, Vesicular glutamate transporter 2; VGLUT3, Vesicular glutamate transporter 3; ZIC2, Zic Family Member 2

REFERENCES

Abraira VE et al. (2017) The Cellular and Synaptic Architecture of the Mechanosensory Dorsal Horn Cell 168:295-310 e219 doi:10.1016/j.cell.2016.12.010

Alba-Delgado C, El Khoueiry C, Peirs C, Dallel R, Artola A, Antri M (2015) Subpopulations of PKCgamma interneurons within the medullary dorsal horn revealed by electrophysiologic and morphologic approach Pain 156:1714-1728 doi:10.1097/j.pain.0000000000000221

Albisetti GW et al. (2019) Dorsal Horn Gastrin-Releasing Peptide Expressing Neurons Transmit Spinal Itch But Not Pain Signals J Neurosci 39:2238-2250 doi:10.1523/JNEUROSCI.2559-18.2019

Aresh B, Freitag FB, Perry S, Blumel E, Lau J, Franck MCM, Lagerstrom MC (2017) Spinal cord interneurons expressing the gastrin-releasing peptide receptor convey itch through VGLUT2-mediated signaling Pain 158:945-961 doi:10.1097/j.pain.0000000000000861

Bardoni R et al. (2019) Pain Inhibits GRPR Neurons via GABAergic Signaling in the Spinal Cord Scientific Reports 9:15804 doi:10.1038/s41598-019-52316-0

Bice TN, Beal JA (1997) Quantitative and neurogenic analysis of neurons with supraspinal projections in the superficial dorsal horn of the rat lumbar spinal cord J Comp Neurol 388:565-574 doi:10.1002/(sici)1096-9861(19971201)388:4<565::aid-cne5>3.0.co;2-0

Bourane S et al. (2015) Identification of a spinal circuit for light touch and fine motor control Cell 160:503-515 doi:10.1016/j.cell.2015.01.011

Boyle KA et al. (2019) Defining a Spinal Microcircuit that Gates Myelinated Afferent Input: Implications for Tactile Allodynia Cell Rep 28:526-540 e526 doi:10.1016/j.celrep.2019.06.040

Boyle KA et al. (2017) A quantitative study of neurochemically defined populations of inhibitory interneurons in the superficial dorsal horn of the mouse spinal cord Neuroscience 363:120-133 doi:10.1016/j.neuroscience.2017.08.044

Cameron D, Polgar E, Gutierrez-Mecinas M, Gomez-Lima M, Watanabe M, Todd AJ (2015) The organisation of spinoparabrachial neurons in the mouse Pain 156:2061-2071 doi:10.1097/j.pain.0000000000000270

Cheng L et al. (2017) Identification of spinal circuits involved in touch-evoked dynamic mechanical pain Nat Neurosci 20:804-814 doi:10.1038/nn.4549

Cui L et al. (2016) Identification of Early RET+ Deep Dorsal Spinal Cord Interneurons in Gating Pain Neuron 91:1413 doi:10.1016/j.neuron.2016.09.010

Del Barrio MG, Bourane S, Grossmann K, Schule R, Britsch S, O'Leary DD, Goulding M (2013) A transcription factor code defines nine sensory interneuron subtypes in the mechanosensory area of the spinal cord PLoS One 8:e77928 doi:10.1371/journal.pone.0077928

Dickie AC et al. (2018) Morphological and functional properties distinguish the substance P and gastrin-releasing peptide subsets of excitatory interneuron in the spinal cord dorsal horn Pain doi:10.1097/j.pain.0000000000001406

Duan B et al. (2014) Identification of spinal circuits transmitting and gating mechanical pain Cell 159:1417-1432 doi:10.1016/j.cell.2014.11.003

Gamboa-Esteves FO, McWilliam PN, Batten TF (2004) Substance P (NK1) and somatostatin (sst2A) receptor immunoreactivity in NTS-projecting rat dorsal horn neurones activated by nociceptive afferent input J Chem Neuroanat 27:251-266 doi:10.1016/j.jchemneu.2004.04.001

Gross MK, Dottori M, Goulding M (2002) Lbx1 specifies somatosensory association interneurons in the dorsal spinal cord Neuron 34:535-549 doi:10.1016/s0896-6273(02)00690-6

Grudt TJ, Perl ER (2002) Correlations between neuronal morphology and electrophysiological features in the rodent superficial dorsal horn J Physiol 540:189-207

Gutierrez-Mecinas M, Bell A, Polgar E, Watanabe M, Todd AJ (2019a) Expression of Neuropeptide FF Defines a Population of Excitatory Interneurons in the Superficial Dorsal Horn of the Mouse Spinal Cord that Respond to Noxious and Pruritic Stimuli Neuroscience 416:281-293 doi:10.1016/j.neuroscience.2019.08.013

Gutierrez-Mecinas M et al. (2017) Preprotachykinin A is expressed by a distinct population of excitatory neurons in the mouse superficial spinal dorsal horn including cells that respond to noxious and pruritic stimuli Pain 158:440-456 doi:10.1097/j.pain.0000000000000778

Gutierrez-Mecinas M, Bell AM, Shepherd F, Polgar E, Watanabe M, Furuta T, Todd AJ (2019b) Expression of cholecystokinin by neurons in mouse spinal dorsal horn J Comp Neurol 527:1857-1871 doi:10.1002/cne.24657

Gutierrez-Mecinas M et al. (2019c) Expression of Calretinin Among Different Neurochemical Classes of Interneuron in the Superficial Dorsal Horn of the Mouse Spinal Cord Neuroscience 398:171-181 doi:10.1016/j.neuroscience.2018.12.009

Gutierrez-Mecinas M, Furuta T, Watanabe M, Todd AJ (2016) A quantitative study of neurochemically defined excitatory interneuron populations in laminae I-III of the mouse spinal cord Mol Pain 12 doi:10.1177/1744806916629065

Gutierrez-Mecinas M, Watanabe M, Todd AJ (2014) Expression of gastrin-releasing peptide by excitatory interneurons in the mouse superficial dorsal horn Mol Pain 10:79 doi:10.1186/1744-8069-10-79

Hantman AW, van den Pol AN, Perl ER (2004) Morphological and physiological features of a set of spinal substantia gelatinosa neurons defined by green fluorescent protein expression J Neurosci 24:836-842

Huang J et al. (2018) Circuit dissection of the role of somatostatin in itch and pain Nat Neurosci doi:10.1038/s41593-018-0119-z

Hughes DI, Sikander S, Kinnon CM, Boyle KA, Watanabe M, Callister RJ, Graham B (2012) Morphological, neurochemical and electrophysiological features of parvalbumin-expressing cells: a likely source of axo-axonic inputs in the mouse spinal dorsal horn J Physiol

Iwagaki N, Garzillo F, Polgar E, Riddell JS, Todd AJ (2013) Neurochemical characterisation of lamina II inhibitory interneurons that express GFP in the PrP-GFP mouse Mol Pain 9:56 doi:10.1186/1744-8069-9-56

Kardon AP et al. (2014) Dynorphin acts as a neuromodulator to inhibit itch in the dorsal horn of the spinal cord Neuron 82:573-586 doi:10.1016/j.neuron.2014.02.046

Kemp T, Spike RC, Watt C, Todd AJ (1996) The mu-opioid receptor (MOR1) is mainly restricted to neurons that do not contain GABA or glycine in the superficial dorsal horn of the rat spinal cord Neuroscience 75:1231-1238 doi:10.1016/0306-4522(96)00333-8

Koch SC et al. (2017) RORbeta Spinal Interneurons Gate Sensory Transmission during Locomotion to Secure a Fluid Walking Gait Neuron 96:1419-1431 e1415 doi:10.1016/j.neuron.2017.11.011

Laing I, Todd AJ, Heizmann CW, Schmidt HH (1994) Subpopulations of GABAergic neurons in laminae I-III of rat spinal dorsal horn defined by coexistence with classical transmitters, peptides, nitric oxide synthase or parvalbumin Neuroscience 61:123-132 doi:10.1016/0306-4522(94)90065-5

Larsson M (2017) Pax2 is persistently expressed by GABAergic neurons throughout the adult rat dorsal horn Neurosci Lett 638:96-101 doi:10.1016/j.neulet.2016.12.015

Levine AJ, Hinckley CA, Hilde KL, Driscoll SP, Poon TH, Montgomery JM, Pfaff SL (2014) Identification of a cellular node for motor control pathways Nat Neurosci 17:586-593 doi:10.1038/nn.3675

Littlewood NK, Todd AJ, Spike RC, Watt C, Shehab SA (1995) The types of neuron in spinal dorsal horn which possess neurokinin-1 receptors Neuroscience 66:597-608 doi:10.1016/0306-4522(95)00039-l

Mar L, Yang FC, Ma Q (2012) Genetic marking and characterization of Tac2-expressing neurons in the central and peripheral nervous system Mol Brain 5:3 doi:10.1186/1756-6606-5-3

Marshall GE, Shehab SA, Spike RC, Todd AJ (1996) Neurokinin-1 receptors on lumbar spinothalamic neurons in the rat Neuroscience 72:255-263 doi:10.1016/0306-4522(95)00558-7

Martin WJ, Liu H, Wang H, Malmberg AB, Basbaum AI (1999) Inflammation-induced up-regulation of protein kinase Cgamma immunoreactivity in rat spinal cord correlates with enhanced nociceptive processing Neuroscience 88:1267-1274

Nelson TS, Fu W, Donahue RR, Corder GF, Hokfelt T, Wiley RG, Taylor BK (2019) Facilitation of neuropathic pain by the NPY Y1 receptor-expressing subpopulation of excitatory interneurons in the dorsal horn Sci Rep 9:7248 doi:10.1038/s41598-019-43493-z

Pagani M, Albisetti GW, Sivakumar N, Wildner H, Santello M, Johannssen HC, Zeilhofer HU (2019) How Gastrin-Releasing Peptide Opens the Spinal Gate for Itch Neuron 103:102-117 e105 doi:10.1016/j.neuron.2019.04.022

Paixão S, Loschek L, Gaitanos L, Alcalà Morales P, Goulding M, Klein R (2019) Identification of Spinal Neurons Contributing to the Dorsal Column Projection Mediating Fine Touch and Corrective Motor Movements Neuron 104:1-16 doi:doi.org/10.1016/j.neuron.2019.08.029.

Peirs C, Patil S, Bouali-Benazzouz R, Artola A, Landry M, Dallel R (2014) Protein kinase C gamma interneurons in the rat medullary dorsal horn: distribution and synaptic inputs to these neurons, and subcellular localization of the enzyme J Comp Neurol 522:393-413 doi:10.1002/cne.23407

Peirs C et al. (2015) Dorsal Horn Circuits for Persistent Mechanical Pain Neuron 87:797-812 doi:10.1016/j.neuron.2015.07.029

Polgar E, Durrieux C, Hughes DI, Todd AJ (2013a) A quantitative study of inhibitory interneurons in laminae I-III of the mouse spinal dorsal horn PLoS One 8:e78309 doi:10.1371/journal.pone.0078309

Polgar E, Fowler JH, McGill MM, Todd AJ (1999) The types of neuron which contain protein kinase C gamma in rat spinal cord Brain Res 833:71-80

Polgar E, Furuta T, Kaneko T, Todd A (2006) Characterization of neurons that express preprotachykinin B in the dorsal horn of the rat spinal cord Neuroscience 139:687-697

Polgar E, Hughes DI, Riddell JS, Maxwell DJ, Puskar Z, Todd AJ (2003) Selective loss of spinal GABAergic or glycinergic neurons is not necessary for development of thermal hyperalgesia in the chronic constriction injury model of neuropathic pain Pain 104:229-239

Polgar E, Sardella TC, Tiong SY, Locke S, Watanabe M, Todd AJ (2013b) Functional differences between neurochemically defined populations of inhibitory interneurons in the rat spinal dorsal horn Pain 154:2606-2615 doi:10.1016/j.pain.2013.05.001

Polgar E, Sardella TC, Watanabe M, Todd AJ (2011) Quantitative study of NPY-expressing GABAergic neurons and axons in rat spinal dorsal horn J Comp Neurol 519:1007-1023

Proudlock F, Spike RC, Todd AJ (1993) Immunocytochemical study of somatostatin, neurotensin, GABA, and glycine in rat spinal dorsal horn J Comp Neurol 327:289-297 doi:10.1002/cne.903270210

Qiu Q, Chen H, Johnson RL (2009) Lmx1b-expressing cells in the mouse limb bud define a dorsal mesenchymal lineage compartment Genesis 47:224-233 doi:10.1002/dvg.20430

Ross SE et al. (2010) Loss of inhibitory interneurons in the dorsal spinal cord and elevated itch in Bhlhb5 mutant mice Neuron 65:886-898 doi:10.1016/j.neuron.2010.02.025

Rowan S, Todd AJ, Spike RC (1993) Evidence that neuropeptide Y is present in GABAergic neurons in the superficial dorsal horn of the rat spinal cord Neuroscience 53:537-545 doi:10.1016/0306-4522(93)90218-5

Sardella TC, Polgar E, Garzillo F, Furuta T, Kaneko T, Watanabe M, Todd AJ (2011a) Dynorphin is expressed primarily by GABAergic neurons that contain galanin in the rat dorsal horn Mol Pain 7:76

Sardella TC, Polgar E, Watanabe M, Todd AJ (2011b) A quantitative study of neuronal nitric oxide synthase expression in laminae I-III of the rat spinal dorsal horn Neuroscience 192:708-720

Simmons DR, Spike RC, Todd AJ (1995) Galanin is contained in GABAergic neurons in the rat spinal dorsal horn Neurosci Lett 187:119-122 doi:10.1016/0304-3940(95)11358-4

Smith KM et al. (2015) Functional heterogeneity of calretinin-expressing neurons in the mouse superficial dorsal horn: implications for spinal pain processing J Physiol 593:4319-4339 doi:10.1113/JP270855

Smith KM, Boyle KA, Mustapa M, Jobling P, Callister RJ, Hughes DI, Graham BA (2016) Distinct forms of synaptic inhibition and neuromodulation regulate calretinin-positive neuron excitability in the spinal cord dorsal horn Neuroscience 326:10-21 doi:10.1016/j.neuroscience.2016.03.058

Solorzano C et al. (2015) Primary afferent and spinal cord expression of gastrin-releasing peptide: message, protein, and antibody concerns J Neurosci 35:648-657 doi:10.1523/JNEUROSCI.2955-14.2015

Spike RC, Puskar Z, Andrew D, Todd AJ (2003) A quantitative and morphological study of projection neurons in lamina I of the rat lumbar spinal cord Eur J Neurosci 18:2433-2448

Spike RC, Puskar Z, Sakamoto H, Stewart W, Watt C, Todd AJ (2002) MOR-1-immunoreactive neurons in the dorsal horn of the rat spinal cord: evidence for nonsynaptic innervation by substance P-containing primary afferents and for selective activation by noxious thermal stimuli Eur J Neurosci 15:1306-1316 doi:10.1046/j.1460-9568.2002.01969.x

Tiong SY, Polgar E, van Kralingen JC, Watanabe M, Todd AJ (2011) Galanin-immunoreactivity identifies a distinct population of inhibitory interneurons in laminae I-III of the rat spinal cord Mol Pain 7:36

Todd AJ, McGill MM, Shehab SA (2000) Neurokinin 1 receptor expression by neurons in laminae I, III and IV of the rat spinal dorsal horn that project to the brainstem Eur J Neurosci 12:689-700 doi:10.1046/j.1460-9568.2000.00950.x

Todd AJ, Russell G, Spike RC (1992) Immunocytochemical evidence that GABA and neurotensin exist in different neurons in laminae II and III of rat spinal dorsal horn Neuroscience 47:685-691 doi:10.1016/0306-4522(92)90176-3

Todd AJ, Spike RC, Polgar E (1998) A quantitative study of neurons which express neurokinin-1 or somatostatin sst2a receptor in rat spinal dorsal horn Neuroscience 85:459-473

Todd AJ, Sullivan AC (1990) Light microscope study of the coexistence of GABA-like and glycine-like immunoreactivities in the spinal cord of the rat J Comp Neurol 296:496-505

Wang D, Tawfik VL, Corder G, Low SA, Francois A, Basbaum AI, Scherrer G (2018) Functional Divergence of Delta and Mu Opioid Receptor Organization in CNS Pain Circuits Neuron 98:90-108 e105 doi:10.1016/j.neuron.2018.03.002

Xu Y et al. (2008) Tlx1 and Tlx3 coordinate specification of dorsal horn pain-modulatory peptidergic neurons J Neurosci 28:4037-4046 doi:10.1523/JNEUROSCI.4126-07.2008

Yasaka T, Tiong SY, Hughes DI, Riddell JS, Todd AJ (2010) Populations of inhibitory and excitatory interneurons in lamina II of the adult rat spinal dorsal horn revealed by a combined electrophysiological and anatomical approach Pain 151:475-488

Yoshida S, Senba E, Kubota Y, Hagihira S, Yoshiya I, Emson PC, Tohyama M (1990) Calcium-binding proteins calbindin and parvalbumin in the superficial dorsal horn of the rat spinal cord Neuroscience 37:839-848 doi:10.1016/0306-4522(90)90113-i

Yuengert R et al. (2015) Origin of a Non-Clarke's Column Division of the Dorsal Spinocerebellar Tract and the Role of Caudal Proprioceptive Neurons in Motor Function Cell Rep 13:1258-1271 doi:10.1016/j.celrep.2015.09.064

Zhang MD et al. (2016) Comparative anatomical distribution of neuronal calcium-binding protein (NECAB) 1 and -2 in rodent and human spinal cord Brain Struct Funct 221:3803-3823 doi:10.1007/s00429-016-1191-3

Zhang MD et al. (2014) Neuronal calcium-binding proteins 1/2 localize to dorsal root ganglia and excitatory spinal neurons and are regulated by nerve injury Proc Natl Acad Sci U S A 111:E1149-1158 doi:10.1073/pnas.1402318111
